# Linear Response of General Observables in Spiking Neuronal Network Models

**DOI:** 10.3390/e23020155

**Published:** 2021-01-27

**Authors:** Bruno Cessac, Ignacio Ampuero, Rodrigo Cofré

**Affiliations:** 1 Biovision Team, INRIA and Neuromod Institute, Université Côte d’Azur, 06902 Sophia Antipolis, France; bruno.cessac@inria.fr; 2 Departamento de Informática, Universidad Técnica Federico Santa María, 2340000 Valparaíso, Chile; ignacio.ampuero.13@sansano.usm.cl; 3 CIMFAV-Ingemat, Facultad de Ingeniería Universidad de Valparaíso, 2340000 Valparaíso, Chile

**Keywords:** neuronal network dynamics, spike train statistics, linear response, non-Markovian dynamics, Gibbs distributions, maximum entropy principle

## Abstract

We establish a general linear response relation for spiking neuronal networks, based on chains with unbounded memory. This relation allow us to predict the influence of a weak amplitude time dependent external stimuli on spatio-temporal spike correlations, from the spontaneous statistics (without stimulus) in a general context where the memory in spike dynamics can extend arbitrarily far in the past. Using this approach, we show how the linear response is explicitly related to the collective effect of the stimuli, intrinsic neuronal dynamics, and network connectivity on spike train statistics. We illustrate our results with numerical simulations performed over a discrete time integrate and fire model.

## 1. Introduction

Neurons communicate by short-lasting electrical signals called action potentials or “spikes”, allowing the rapid propagation of information throughout the nervous system, with a minimal energy dissipation [1]. The spike shape is remarkably constant for a given neuron, and it is a contemporary view to consider spikes as quanta (bits) of information [2]. As a result, information is presumably encoded in the spike timing [3].

**Figure 1:**
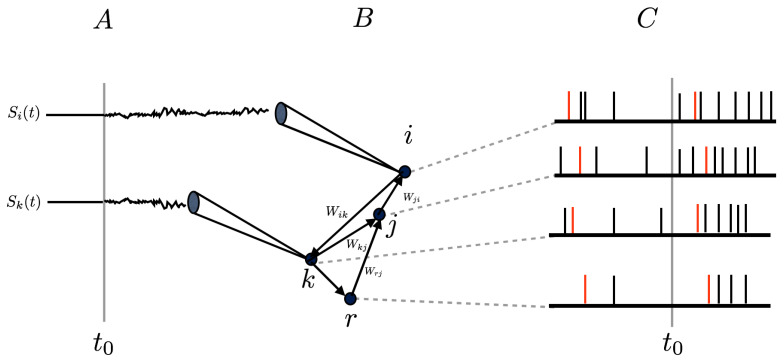
A time-dependent stimulus (A) is applied at time $t_{0}$ to some neurons of a spiking neuronal network (B). As a result, the spiking activity is modified as well as spike correlations between neurons (C), even for neurons not directly stimulated, because of direct or indirect synaptic interactions. These are represented in the figure by the weights W.

The simplest quantitative way to characterize the spiking activity of a neuron is its firing rate r(t), where r(t)dt is the probability that this neuron spikes during a small interval [t, t+dt]. Under the influence of an external stimulus the firing rate changes. A classical ansatz, coming from the Volterra expansions [2] is to write the variation in the firing rate of a neuron as the convolution form:

(1) $$\delta^{\left(1\right)}\left[r\left(t\right)\right]=\left(K*S\right)\left[t\right],$$

where the exponent ${}^{\left(1\right)}$ recalls that we consider a first-order effect of the stimulus *S*, that is, the stimulus is weak enough so that higher-order terms in the Volterra expansion can be neglected. This is an example of linear response: the variation in the rate is proportional to the stimulus. Here, *K* is a convolution kernel constrained by the underlying network dynamics. For example, in sensory neurons, where *S* and *K* are functions of space and time, the convolution (1) takes the explicit form:

(2) $$\left(K*S\right)\left[t\right]=\int_{x=-\infty }^{+\infty }\int_{y=-\infty }^{+\infty }\int_{\tau =-\infty }^{t}K\left(x,y,t-\tau \right)S\left(x,y,\tau \right)d\tau dxdy,$$

where *K* decays sufficiently fast at infinity (in space and time) to ensure that the integral is well defined. *K* mimics the receptive field (RF) of the neuron. In general, the response of spiking neuronal networks to a time-dependent stimulus does not only affect rates, it also has an impact on higher-order correlations between neurons, because neurons are connected through synapses. This situation is sketched in Figure 1.

In particular, sensory neurons collectively convey to the brain information about external stimuli using correlated spike patterns resulting from the conjunction of stimulus influence, intrinsic neurons dynamics and neurons interactions via synapses [4,5,6,7,8]. This correlated firing has been linked to stimulus encoding [9], stimulus discrimination [10,11] and to intrinsic properties of the network which remain in absence of stimulus [12]. However, disentangling the biophysical origins of the correlations observed in spiking data is still a central and difficult problem in neuroscience [2,13,14]. As a consequence, correlations in spiking neuronal networks have attracted a considerable amount of attention in the last years, from experimental data analysis perspectives [4,6,7,15] as well as from the theoretical modeling viewpoint [1,16,17,18,19,20].

On one hand, novel experimental recording techniques such as Multi-Electrode Arrays (MEA) permit the measurement of the collective spiking activity of larger and larger populations of interacting neurons responding to external stimuli [21,22]. These recordings allow, in particular, for better characterizations of the link between stimuli and the correlated responses of a living neuronal network, paving the way to better understand “the neural-code” [2]. Yet, neuronal responses are highly variable [13]. Even at the single neuron level, when presenting repetitions of the same stimulus under controlled experimental conditions, the neural activity changes from trial to trial [23,24]. Thus, researchers are seeking statistical regularities in order to unveil a probabilistic, causal, relationship between stimuli and spiking responses [4,5,7,25,26,27].

On the other hand, mathematical models of spiking neuronal networks offer a complementary approach to biological experiments [13,14,28,29]. Based on bio-physically plausible mechanisms describing the dynamics of neurons, mathematical modeling provides a framework to characterize the population spike train statistics in terms of biophysical parameters, synaptic connectivity, history of previous spikes and stimuli. The hope is that understanding these aspects in a model may help to better the processing and extraction of information from real data.

In this article, we consider a spiking neuronal network model where the non-linear dynamics and neurons interactions naturally produce spatio-temporal spikes correlations. We assume that these neurons reach a stationary state without stimuli and from a given time are submitted to a time-dependent stimulation (See Figure 1). How are the spatio-temporal spike correlations modified by this stimulus? We address this question in the context of linear response theory using methods from ergodic theory and so-called chains with complete connections [30], extending the notion of Markov chains to infinite memory, providing a generalized notion of Gibbs distribution. We show that spatio-temporal response is written in terms of a history-dependent convolution kernel applied to the stimuli, generalizing (2) to more general observables than rates. We compute explicitly this kernel in a specific model example and analyze the validity of our linear response formula by numerical means.

The linear response determines how the expectation value of an observable of a dynamical system changes upon weakly perturbing the dynamics. The response of a system, originally at equilibrium, to a time-dependent stimulus is proportional to the stimulus, with coefficients obtained via correlation functions computed at equilibrium. The theory we develop has its roots in non-equilibrium statistical physics (briefly reviewed in Section 2.1). A seminal result in this context is the fluctuation–dissipation theorem [31], where the linear response only depends on the correlation functions of the unperturbed system. Our approach proceeds along similar lines, meaning that the linear response can be predicted from the spikes correlations of the unperturbed system (stationary state). We derive a result of this type for a discrete-time integrate and fire model addressing a central question: which correlations matter and how they are related to synaptic interactions? Among the applications of our results is the characterization of population receptive fields in terms of the underlying synaptic connectivity, non-linear dynamics and background activity, the prediction of higher-order correlations in the population responses to non-stationary weak amplitude stimuli, assessing the role of the synaptic connectivity in motion processing from the spiking response to, etc.

The paper is organized as follows. In Section 2, we briefly review linear response in statistical physics and ergodic theory allowing us to make a link between neuronal networks, considered as dynamical systems, and the statistics of spikes. We introduce the formalism of chains with unbounded memory (which are, as we explain, equivalent to left-sided one-dimensional Gibbs distributions), allowing the handling of non-stationary spike distribution with unbounded memory. In Section 3, we derive the general linear response formula (16) used throughout the paper. This equation expresses the time-dependent variation in the average of an observable *f* as a time series of specific correlation functions computed with respect to spontaneous activity (without stimulus). This result, reminiscent of the fluctuation-dissipation theorem in statistical physics [32,33], is applied here to spike statistics. In Section 4 we introduce a spiking neuronal network model to instantiate our analysis. This model has been presented in [34]. We associate the spiking activity to a discrete stochastic process defined from transition probabilities where memory is unbounded. These probabilities are written as a function of the parameters of the model. We explicitly wrote a discrete-time form of the convolution kernel (1) as an explicit function of the model parameters, especially synaptic weights. The expression relies on a Markovian approximation of the chain and on a decomposition theorem of spike observables, introduced in a more general context by Hammersley and Clifford in 1971 [35] (see Section 2.2). In Section 5 we illustrate through an example our linear response theory applied. In particular, we show how one can predict the variation in the firing rate and in the delayed pairwise correlation between two neurons from the mere knowledge of the stimulus and relevant spontaneous correlations. Finally, in Section 6 we discuss our results.

## 2. Linear Response, Gibbs Distributions and Probabilistic Chains with Unbounded Memory

Neuronal networks can be considered either as dynamical systems (when the dynamics is known) or as spike generating processes characterized by transition probabilities computed from spike train observations. In the first case, it is natural to seek a linear response from dynamics itself, using approximations (e.g., mean-field [36]). In the second case, one has to define a probability distribution on the spike trains in order to investigate the effect of a perturbation. In this section, we show how these two approaches are related, making a link between the classical statistical physics approach of linear response, dynamical systems and ergodic theory, and neuronal networks. We introduce then the general formalism of chains with unbounded memory allowing the handling of non-equilibrium linear response for spiking neuronal networks. All of the material in this section is known in different domains, statistical physics, ergodic theory, stochastic processes, neuronal networks, and is presented here for a better understanding of the next sections.

### 2.1. Linear Response in Statistical Physics

For simplicity, we consider in this introductory section a dynamical system taking a finite number of “configurations" denoted by ω. In statistical physics, the linear response theory can be addressed in these terms. In a system at thermodynamic equilibrium, the probability of a configuration ω is given by the Boltzmann–Gibbs distribution:

(3) $$\mu \left[\omega \;\right]=\frac{1}{Z}e^{-\frac{H\left(\omega \;\right)}{k_{B}T}},$$

where $Z=\sum_{\omega }e^{-\frac{H\left(\omega \;\right)}{k_{B}T}}$ is called the partition function, with $k_{B}$, the Boltzmann constant and *T* the temperature. The function $H\left(\omega \;\right)$ is called the energy

(4) $$H\left(\omega \;\right)=\sum_{\alpha }\lambda_{\alpha }X_{\alpha }\left(\omega \;\right).$$

The functions $X_{\alpha }$ are extensive quantities (proportional to the number of particles) such as energy, electric charge, volume, number of particles, magnetic field, … The conjugated parameters $\lambda_{\alpha }$ correspond to intensive quantities (not proportional to the number of particles), like temperature, electric potential, pressure, chemical potential, magnetic susceptibility, …. In general, they depend on the location in the physical space (e.g., the temperature depends on the position in a fluid). At equilibrium, they are uniform in space though. The form of *H*, i.e., the choice of the $\lambda_{\alpha }$ and $X_{\alpha }$ is constrained by the physical properties of the system. It is also constrained by boundary conditions.

In standard statistical physics courses, the Gibbs distribution form (3) is obtained as a consequence of the Maximum Entropy Principle [37]. For a probability measure *P* on the set of configurations, the statistical entropy is:

(5) $$S\left[P\;\right]=-k_{B}\sum_{\omega }\log P\left[\omega \;\right]\log P\left[\omega \;\right].$$

Denote $E_{P}\left[\;\;\right]$ the expectation with respect to *P*. The Maximum Entropy Principle seeks a probability distribution maximizing the statistical entropy under the constraint that the average energy is constant, i.e., $E_{P}\left[\;H\;\right]=C$ for any probability measure *P* on the set of configurations. This probability exists and is unique when the set of configurations is finite; this is (3). When this set is infinite (e.g., thermodynamic limit) additional summability conditions are required on *H* to ensure existence and uniqueness of *P* [38,39].

A non-equilibrium situation arises when the $\lambda_{\alpha }$s are not uniform in space, generating gradients $\vec{\nabla }\lambda_{\alpha }$ (temperature gradient, electric potential gradient…). These gradients result in currents of $X_{\alpha }$ (e.g., a temperature gradient induces a heat current). In general, the currents are nonlinear functions of gradients. The Onsager linear response theory assumes that currents are linear combinations of gradients (i.e., gradients are weak enough so that non-linear terms can be neglected). Known examples are Ohm’s law where the electric current is proportional to the gradient of the electric potential, Fourier’s law where the heat flux is proportional to the temperature gradient, Fick’s law etc. Several gradients can be simultaneously involved like in the Peltier effect. The proportionality coefficients are called Onsager coefficients [40].

Now, the property that interests us is that Onsager coefficients are obtained as correlations functions computed at equilibrium (Kubo relations [32]). Thus, the knowledge of correlations at equilibrium allows the inference of the non-equilibrium response of the system to weak perturbations.

### 2.2. Linear Response in Spiking Neuronal Networks

Neuronal networks are modeled by dynamical systems (possibly stochastic). Therefore, the linear response theory can be addressed using the tools briefly presented in the previous section. This has been done for discrete-time in the Amari–Wilson–Cowan model [41,42], where the convolution kernel *K* appearing in (2) can be explicitly computed [43,44]. Notably, one can compute the response of a neuron to a weak harmonic perturbation of another neuron, exhibiting specific resonances and functional connectivity distinct from the synaptic graph.

When dealing with spiking models such as the Integrate and Fire, the dynamics are not differentiable anymore (because of the mechanism of reset at threshold). Still, the Markov chain formalism (and its extension to infinite memory) can be used. In particular, we exhibit an example where transition probabilities can be explicitly computed and directly related to the dynamics.

We consider a network of *N* neurons, labeled by the index k=1…N. We define a spike variable $\omega_{k}\left(n\right)=1$ if neuron *k* has emitted a spike in the time interval [nδ, (n+1)δ], and $\omega_{k}\left(n\right)=0$ otherwise. We denote by $\omega \left(n\right):={\left[\omega_{k}\left(n\right)\;\right]}_{k=1}^{N}$ the spike-state of the entire network at time *n*, which we call a spiking pattern. We denote by $A={\left\{\;0,1\;\right\}}^{N}$, the state space of spiking patterns in a network of *N* neurons; a spike block denoted by $\omega_{m}^{n}$, n≥m, is the sequence of spike patterns ω(m), ω(m+1),…, ω(n); blocks are elements of the product set $A^{n-m}$ also denoted $A_{m}^{n}$ in the text. We use this last notation because we consider processes with infinite memory (m→−∞) and we want to have an explicit notation $A_{-\infty }^{n}$ for the corresponding set of events. The time-range (or “range”) of a block $\omega_{m}^{n}$ is n−m+1, the number of time steps from *m* to *n*. We call a spike train an infinite sequence of spikes both in the past and in the future. The set of spike trains is thus $\Omega \equiv A^{Z}$. To simplify notations we note a spike train ω∈Ω. The shift operator σ:Ω→Ω is $\sigma \omega =\omega^{\prime }$, with $\omega^{\prime }\left(n\right)=\omega \left(n+1\right)$. This allow us to go one step forward in time along the spike train ω.

We note $F_{\leq n}$ the set of measurable events (filtration) before time *n* and F the filtration on Ω. P(Ω,F) is the set of probability measures on (Ω,F).

We use the notion of (spike) observable. This is a function f:Ω→R that associates a real number to a spike-train. We say that the observable f:Ω→R has range R=D+1 if $f\left(\omega \right)\equiv f\left(\omega_{0}^{D}\right)$. It follows from the Hammersley–Clifford theorem [35,45] that any range-*R* observable can be written in the form:

(6) $$f\left(\omega \right)=\sum_{l}f_{l}\;m_{l}\left(\omega \right),$$

where $f_{l}$ are real numbers, the coefficients of the decomposition of *f* in the finite space of range *R*-observables. The functions $m_{l}$ spanning this space are called monomials [8]. They have the form:

$$m_{l}\left(\omega \right)=\prod_{k=1}^{n}\omega_{i_{k}}\left(t_{k}\right).$$

where $i_{k}=1\ldots N$ is a neuron index, and $t_{k}=0\ldots D$. Thus, $m_{l}\left(\omega \right)=1$ if and only if, in the spike train ω, neuron $i_{1}$ spikes at time $t_{1}$, …, neuron $i_{k}$ spikes at time $t_{k}$. Otherwise, $m_{l}\left(\omega \right)=0$. The number *n* is the degree of the monomial; degree one monomials have the form $\omega_{i_{1}}\left(t_{1}\right)$, degree two monomials have the form $\omega_{i_{1}}\left(t_{1}\right)\omega_{i_{2}}\left(t_{2}\right)$, and so on. Thus, monomials are similar to what physicists call (spike) interactions; in our case these interactions involve a time delay between spikes. There are $L=2^{NR}$ monomials of *N* neurons and range *R* and one can index each of them by an integer *l* in one-to-one correspondence with the set of pairs $\left(i_{k},t_{k}\right)$ (see Equation (A2) in the Appendix A). The advantage of monomial representation is to focus on spike events, which is natural for spiking neuronal dynamics.

We now introduce time-dependent observables. These are functions f(t,ω) depending on time *t* (continuous or discrete) and on the spike train ω. The notation f(t,ω) stands here for $f(t,\omega_{-\infty }^{\left[\;t\;\right]})$ where $\left[\;t\;\right]$ is the integer part of *t*: the function depends on the spike train ω via spikes preceding the current time *t*. This is an implementation of causality. A range-*R* time dependent observable is a function $f\left(t,\omega \right)\equiv f\left(t,\omega_{\left[\;t\;\right]-D}^{\left[\;t\;\right]}\right)$. The decomposition (6) also holds for a time dependent range-*R* observable

$$f\left(t,\omega \right)=\sum_{l}f_{l}\left(t\right)\;m_{l}\left(\sigma^{\left[\;t\;\right]}\omega \right),$$

where $f_{l}\left(t\right)$ are now functions of time, and $\sigma^{\left[\;t\;\right]}$ is the $\left[\;t\;\right]$-iterate of the time shift operator σ.

### 2.3. Homogeneous Markov Chains and Gibbs Distributions

A “natural” way to characterize the statistics of observed spike trains is to associate them to a Markov chain with transition probabilities $P_{n}\left[\omega \left(n\right)\;\left|\;\omega_{n-D}^{n-1}\right.\;\right]$, where the index *n* in $P_{n}$ indicates that the transition probabilities depend on time *n*. This approach is “natural” because it captures causality by conditioning on the past spikes. We call *D* the memory depth of the chain and set R=D+1.

#### 2.3.1. Invariant Probability

Let us start the discussion when transition probabilities are independent of time (homogeneous Markov chain). In this case, we drop the index *n* in the transition probabilities, $P\left[\omega \left(n\right)\;\left|\;\omega_{n-D}^{n-1}\right.\;\right]$. Assuming that all transition probabilities are strictly positive, it follows from the Perron-Frobenius theorem [46,47] that the Markov chain has a unique invariant probability *p* on $A^{D}$. From the Chapman–Kolmogorov equation [46] one constructs, from *p* and transition probabilities, a probability measure μ on P(Ω,F). where:

(7) $$\mu \left[\omega_{m}^{n}\;\right]\;=\prod_{l=m+D}^{n}P\left[\omega \left(l\right)\;\left|\;\omega_{l-D}^{l-1}\right.\;\right]\;p\left[\omega_{m}^{m+D-1}\;\right],\;\forall m<n\in Z.$$

As we now discuss, there is a natural correspondence between μ and exponential distributions of the form (4) (Gibbs distributions).

#### 2.3.2. Transfer Matrix

Let us now consider a range-*R* observable:

(8) $$H\left(\omega \;\right)\;=\sum_{l}h_{l}\;m_{l}\left(\omega \right),$$

where $h_{l}>C>-\infty$. Any block $\omega_{0}^{D}$ of range R=D+1 can be viewed as a transition from a block $\varpi^{\left(u\right)}=\omega_{0}^{D-1}$ to the block $\varpi^{\left(u^{\prime }\right)}=\omega_{1}^{D}$. We write $\omega_{0}^{D}∼\varpi^{\left(u\right)}\varpi^{\left(u^{\prime }\right)}$. By extension, for two blocks $\varpi^{\left(u\right)},\varpi^{\left(u^{\prime }\right)}$ of range D≥1 we say that the transition $\varpi^{\left(u\right)}\to \varpi^{\left(u^{\prime }\right)}$ is legal if there is a block $\omega_{0}^{D}∼\varpi^{\left(u\right)}\varpi^{\left(u^{\prime }\right)}$. On this basis, one can construct a transfer matrix with positive entries:

$$L_{\varpi^{\left(u\right)},\varpi^{\left(u^{\prime }\right)}}=\;\left\{\begin{array}{cc} e^{H(\omega_{0}^{D})},\; & \mathrm{if}\omega_{0}^{D}∼\varpi^{\left(u\right)}\varpi^{\left(u^{\prime }\right)};\\ 0,\; & \mathrm{otherwise}. \end{array}\right.$$

It follows from the Perron-Frobenius theorem [46,47] that L has a unique real positive eigenvalue *s*, strictly larger in modulus than the other eigenvalues, and with positive right eigenvector LR=sR, and left eigenvector LL=sL. Moreover, the range-*R* observable:

$$\phi \left(\omega_{0}^{D}\right)=H\left(\omega_{0}^{D}\right)-\log R\left(\omega_{0}^{D-1}\;\right)+\log R\left(\omega_{1}^{D}\;\right)-\log s$$

defines an homogeneous Markov chain [48] with transition probabilities $P\left[\omega \left(D\right)\;\left|\;\omega_{0}^{D-1}\right.\;\right]=e^{\phi (\omega_{0}^{D})}$.

#### 2.3.3. Invariant Probability and Gibbs Distribution

The unique invariant probability of this Markov chain is:

$$p\left(\omega_{0}^{D-1}\right)=R\left(\omega_{0}^{D-1}\;\right)L\left(\omega_{0}^{D-1}\;\right).$$

Using the Chapman–Kolmogorov Equation (7) one extends *p* to a probability μ on Ω, where, for m+n>D:

(9) $$\mu \left[\omega_{m}^{n}\;|\;\omega_{m-D-1}^{m-1}\;\right]\;=\frac{e^{\sum_{l=m-D}^{n-D}H\left(\omega_{l}^{l+D}\;\right)}R\left(\omega_{n-D+1}^{n}\;\right)L\left(\omega_{m-D}^{m-1}\;\right)}{s^{n-m+1}}.$$

This emphasizes the Markovian nature of the process since the conditioning has a finite time horizon of depth *D*.

It follows therefore that the probability of observing a spike block $\omega_{m}^{n}$, given a certain past $\omega_{m-D-1}^{m-1}$ is proportional to $e^{\sum_{l=m-D}^{n-D}H\left(\omega_{l}^{l+D}\;\right)}$. If *H* is formally interpreted as an energy then $\sum_{l=m-D}^{n-D}H\left(\omega_{l}^{l+D}\;\right)$ is the energy of the block $\omega_{m}^{n}$. Note that, in contrast to (4) we have removed the − sign which has no reason to be present in this context, and is a source of nuisance when doing computations.

This establishes a first relationship with Gibbs distributions of the form (3), with a strong difference though. More precisely one has, ∃A,B>0 such that, for any block $\omega_{0}^{n}$,

$$A\leq \frac{\mu \left[\omega_{0}^{n}\;\right]}{e^{-(n-D+1)P(H)}e^{-\sum_{k=0}^{n-D}H\left(\omega_{k}^{k+D}\;\right)}}\leq B,$$

which defines, in ergodic theory, a Gibbs measure in the sense of Bowen [49]. Whereas we assumed (3) to hold on a finite set of states characterizing the system at a given time, here ω is a trajectory of the system describing its time evolution. In addition, the probability of the block $\omega_{m}^{n}$ is conditioned upon the past, which, in statistical physics would correspond to determine the probability of a block of binary variables (say spins) $\omega_{m}^{n}$ with left boundary conditions $\omega_{m-D-1}^{m-1}$. This analogy is further developed in the next section.

In the case of a Markov chain, the entropy (5) extends to:

$$S\left(\mu \right)=-\sum_{\omega_{0}^{D}}p\left(\omega_{0}^{D-1}\;\right)P\left[\omega \left(D\right)\;\left|\;\omega_{0}^{D-1}\right.\;\right]\log P\left[\omega \left(D\right)\;\left|\;\omega_{0}^{D-1}\right.\;\right],$$

where we have dropped the Boltzmann constant as it plays no role here. Then, it can be shown that μ satisfies a variational principle [48,49]; it maximizes $S\left[\nu \;\right]\;+\;E_{\nu }\left[\;H\;\right]$, where ν is an invariant probability on P(Ω,F). If $E_{\nu }\left[\;H\;\right]$ is fixed this amounts to maximizing the entropy under the constraint that the average energy $E_{\nu }\left[\;H\;\right]$ is fixed. Finally, the supremum $F=S\left[\nu \;\right]\;+\;E_{\nu }\left[\;H\;\right]$ corresponds to the free energy; the generating function of cumulants.

We have therefore shown that a potential of the form (8) is associated with a homogeneous Markov chain where the invariant probability, extends the notion of Gibbs distribution, introduced in Section 2.1, to systems with memory where the probability to be in a state depends on a finite history. The extension to infinite history is made in the next section. As an important remark, the detailed balance condition is not required to define Gibbs distributions from Markov chains.

### 2.4. Chains with Infinite Memory and Gibbs Distributions

In the previous section we have made two important assumptions: (i) memory is bounded (finite memory depth *D*); (ii) the correspondence between Markov chains and Gibbs form was established for homogeneous Markov chains.

However, when considering neural networks, the memory may neither constant nor bounded: consider for example an Integrate and Fire model where the memory goes back to the last time in the past when the neuron has fired; in general, it is not possible to bound this time. So, the most general formalism is to consider chains with unbounded memory [50,51,52]. Of course, as we discuss below, Markovian approximations are possible and useful. Still, one needs to properly control these approximations. In addition, we want to consider here the case of a system submitted to a time-dependent stimulus, where the dynamic is not time-translation invariant.

Thus, we are now considering a family of transition probabilities of the form $P_{n}\left[\omega \left(n\right)\;\left|\;\omega_{-\infty }^{n-1}\right.\;\right]$, which represent the probability, that at time *n*, one observes the spiking pattern ω(n) given the (unbounded) network spike history. Such a non-Markovian stochastic process is known as a “chain with complete connections” or a “chain with unbounded memory” [30] defined in more detail here. This section follows very closely from [53].

**Definition** **1.** *A system of transition probabilities is a family ${\left\{P_{n}\right\}}_{n\in Z}$ of functions with $P_{n}\left[\cdot \;\left|\;\cdot \right.\;\right]:A\times A_{-\infty }^{n-1}\to \left[0,1\right]$ such that the following conditions hold for every n∈Z:*

***(a)***  *For every ω(n)∈A the function $P_{n}\left[\omega \left(n\right)\;\left|\;\cdot \right.\;\right]$ is measurable with respect to $F_{\leq n-1}$.*
***(b)***  *For every $\omega_{-\infty }^{n-1}\in A_{-\infty }^{n-1}$,*
  $$\sum_{\omega (n)\in A}P_{n}\left[\omega \left(n\right)\;\left|\;\omega_{-\infty }^{n-1}\right.\;\right]\;=1.$$

**Definition** **2.** *A probability measure μ in P(Ω,F) is consistent with a system of transition probabilities ${\left\{P_{n}\right\}}_{n\in Z}$ if:*

(10) $$\int h\left(\omega_{-\infty }^{n}\right)\mu \left(d\omega \right)=\int \sum_{\omega (n)\in A}h\left(\omega_{-\infty }^{n-1}\omega \left(n\right)\right)P_{n}\left[\omega \left(n\right)\;\left|\;\omega_{-\infty }^{n-1}\right.\;\right]\mu \left(d\omega \right),$$

*for all n∈Z and all $F_{\leq n}$-measurable functions h. The probability measure μ, when it exists, is called a chain with complete connections consistent with the system of transition probabilities ${\left\{P_{n}\right\}}_{n\in Z}.$ It is possible that multiple measures are consistent with the same system of transition probabilities.*

We now give conditions ensuring the existence of a probability measure consistent with the system of transition probabilities [53].

**Definition** **3.** *A system of transition probabilities is non-null on Ω if, for all n∈Z and all $\omega_{-\infty }^{n}\in A_{-\infty }^{n}$:*

$$P\left[\omega \left(n\right)\;\left|\;\omega_{-\infty }^{n-1}\right.\;\right]\;>0$$

*We note, for n∈Z, m≥0, and r integer:*

$$\omega \overset{m,n}{=}\omega^{\prime },\;if\;\omega \left(r\right)=\omega^{\prime }\left(r\right),\forall r\in \left\{n-m,\ldots ,n\right\}.$$

**Definition** **4.** *Let m be a positive integer. The m-variation of $P_{n}\left[\omega \left(n\right)\;\left|\;\cdot \right.\;\right]$ is:*

(11) $$var_{m}\left[P_{n}\left[\omega \left(n\right)\;\left|\;\cdot \right.\;\right]\right]=\sup\left\{|P_{n}\left[\omega \left(n\right)\;\left|\;\omega_{-\infty }^{n-1}\right.\;\right]-P_{n}\left[\omega \left(n\right)|\omega_{-\infty }^{\prime n-1}\right]|:\omega \overset{m,n}{=}\omega^{\prime }\right\}$$

**Definition** **5.** *The function $P_{n}\left[\omega \left(n\right)\;\left|\;\cdot \right.\;\right]$ is continuous if $var_{m}\left[P_{n}\left[\omega \left(n\right)\;\left|\;\cdot \right.\;\right]\right]\to 0$ as m→+∞.*

The intuitive meaning of continuity is the following. The quantity $var_{m}\left[P_{n}\left[\omega \left(n\right)\;\left|\;\cdot \right.\;\right]\right]$ corresponds to the maximum variation one can observe on the probability of the spike state at time *n*, given that the history is fixed up to time n−m. Thus, continuity implies that this variation tends to zero as *m* tends to infinity: the further in the past that the spike sequence is fixed, the smaller the probability that the past influences the present.

The following result holds (see [53]):

**Theorem** **1.** *A system of continuous transition probabilities on a compact space has at least one probability measure consistent with it.*

Uniqueness requires additional technical assumptions [53]. These conditions hold in the discrete time integrate and fire model [34] considered in Section 4.

Let us now elaborate on the link with Gibbs distributions. First, we define $\phi \left(n,\omega \;\right):Z\times \Omega \to R$ by:

(12) $$\phi \left(n,\omega \;\right)\;\equiv \log P\left[\omega \left(n\right)\;\left|\;\omega_{-\infty }^{n-1}\right.\;\right],$$

and:

(13) $$\Phi \left(m,n,\omega \right)=\sum_{r=m}^{n}\phi \left(r,\omega \;\right).$$

Then:

(14) $$P\left[\omega_{m}^{n}\;\left|\;\omega_{-\infty }^{m-1}\right.\;\right]=e^{\Phi (m,n,\omega )}=e^{\sum_{r=m}^{n}\phi \left(r,\omega \;\right)}$$

and:

(15) $$\mu \left[\omega_{m}^{n}\right]=\int_{A_{-\infty }^{m-1}}e^{\Phi (m,n,\omega )}\mu \left(d\omega \right).$$

These equations emphasize the connection with Gibbs distributions in statistical physics where ϕ acts as an “energy” [53,54]. From now on we use the term “potential" instead. The correspondence in our case is to consider “time” as a 1-dimensional “lattice” and the “boundary conditions” as the past $\omega_{-\infty }^{m-1}$ of the stochastic process. In contrast to statistical physics, and because the potential is defined via transition probabilities, the normalization factor (partition function) is equal to 1. For this reason, we call ϕ a normalized Gibbs potential.

Equations (14) and (15) are similar to (9) with an essential difference: the memory is now infinite, and the potential ϕ has an infinite range. As it is well-known in statistical physics [38,39], infinite range potentials require specific conditions to be associated with a unique Gibbs distribution. There is a mathematically well-founded correspondence between chains with complete connections and Gibbs distributions [38,39,53]. However, while chains with complete connections define transition probabilities where the present is conditioned upon the past, Gibbs distributions allow conditioning “upon the future” as well. More generally, Gibbs distributions in statistical physics extend to probability distributions on $Z^{d}$ where the probability (3) to observe a certain configuration of spins in a restricted region of space is constrained by the configuration at the boundaries of this region. They are therefore defined in terms of specifications [38,39], which determine finite-volume conditional probabilities when the exterior of the volume is known. In one spatial dimension (d=1), identifying Z with a time axis, this corresponds to conditioning both in the past and in the future. In contrast, families of transition probabilities with an exponential continuity rate define the so-called left-interval specifications (LIS) [53,55]. This leads to not equivalent notions of “Gibbsianness" [56]. We shall not develop on these distinctions here and call Gibbs distribution a chain with the complete connection.

## 3. Linear Response for Neuronal Networks with Unbounded Memory

We consider a neural system where spike statistics is characterized by a time-translation invariant Gibbs distribution (chain with unbounded memory) $\mu^{\left(sp\right)}$ where “sp” stands for “spontaneous". That is, we suppose that, in the absence of a stimulus, the spontaneous dynamics is stationary. We assume that a stimulus S(t) is applied from time $t=t_{0}$, and that conditions of existence and uniqueness of a chain with complete connection μ are fulfilled in the presence of the stimulus (an example is given in the next section). We note $n_{0}=\;\left[\;t_{0}\;\right]$. For times anterior to $n_{0}$, μ identifies with $\mu^{\left(sp\right)}$, that is, for any $m<n\leq n_{0}$, for any block $\omega_{m}^{n}$, $\mu \left[\omega_{m}^{n}\;\right]\;=\mu^{\left(sp\right)}\left[\omega_{m}^{n}\;\right]$. In contrast, for $n>n_{0}$ spike statistics is modified. Consider a range-*R* observable f(t,ω) then $E_{\mu }\left[\;f(t,.)\;\right]\;\overset{\mathrm{def}}{=}E_{\mu^{\left(sp\right)}}\left[\;f(t,.)\;\right]\;+\;\delta \left[f(t,.)\;\right]$ where $\delta \left[f(t,.)\;\right]\;=0$ for $t<t_{0}$ and $\delta \left[f(t,.)\;\right]\;\neq 0$ for $t\geq t_{0}$.

The goal is to establish an explicit (formal) equation for $\delta \left[f(t,.)\;\right]$, as a function of the stimulus. This is done via a Volterra-like expansion in powers of the stimulus, cut to the first order so as to obtain a linear response in terms of a convolution between the stimulus and a convolution kernel $K_{f}$, depending on *f*, $\delta^{\left(1\right)}\left[f(t,.)\;\right]\;=\;\left[K_{f}*S\;\right]\left(t\right)$. This way, we obtain a relationship between the proportionality coefficient $K_{f}$ in the linear response, and specific correlation functions computed at equilibrium (spontaneous activity). This provides a Kubo relation holding in the case of neuronal networks with unbounded memory and for an arbitrary range-*R* observable *f*. In contrast to Volterra expansion, our formalism allows to explicit the dependence of $K_{f}$ in the neuronal network characteristics (parameters fixing the individual neuron dynamics and connectivity-synaptic weights). An example is provided in the next section.

### 3.1. First Order Expansion

We assume that the statistics of spikes is described by time-dependent chains with unbounded memory, with potential ϕ(n,ω). We note $\delta \phi \left(n,\omega \right)=\phi \left(n,\omega \right)-\phi^{\left(sp\right)}\left(\omega \;\right)$. We define likewise $\Phi \left(n,\omega \right)=\Phi^{\left(sp\right)}\left(\omega \;\right)\;+\;\delta \Phi \left(n,\omega \right)$ using (13).

From the definition (12), $e^{\phi^{\left(sp\right)}}$ corresponds to the family of transition probabilities $\left\{\;P^{\left(sp\right)}\;\right\}$ defining the Gibbs distribution $\mu^{\left(sp\right)}$ in the spontaneous regime, whereas $e^{\phi }$ corresponds to the family of transition probabilities $\left\{\;P\;\right\}$ defining the Gibbs distribution μ in time-dependent stimuli-evoked regime. For $n>n_{0}$, we have:

$$e^{\Phi \left(n_{0}+1,n,\omega \;\right)}=e^{\Phi^{\left(sp\right)}\left(n_{0}+1,n,\omega \;\right)+\delta \Phi \left(n_{0}+1,n,\omega \;\right)}=e^{\Phi^{\left(sp\right)}\left(n_{0}+1,n,\omega \;\right)}\;\left[1+\sum_{p=1}^{+\infty }\frac{\delta \Phi {\left(n_{0}+1,n,\omega \right)}^{p}}{p!}\;\right],$$

which, from Equation (13), gives:

$$P\left[\omega_{n_{0}+1}^{n}\;\left|\;\omega_{-\infty }^{n_{0}}\right.\;\right]=P^{\left(sp\right)}\left[\omega_{n_{0}+1}^{n}\;\left|\;\omega_{-\infty }^{n_{0}}\right.\;\right]\;\left[1+\sum_{p=1}^{+\infty }\frac{\delta \Phi {\left(n_{0}+1,n,\omega \right)}^{p}}{p!}\;\right].$$

Taking the first-order approximation of the exponential, we obtain:

$$e^{\Phi \left(n_{0}+1,n,\omega \;\right)}∼e^{\Phi^{\left(sp\right)}\left(n_{0}+1,n,\omega \;\right)}\;\left[1\;+\;\delta \Phi (n_{0}+1,n,\omega )\;\right].$$

However, while $\Phi \left(n_{0}+1,n,\omega \;\right)$ and $\Phi^{\left(sp\right)}\left(n_{0}+1,n,\omega \;\right)$ are normalized potentials, i.e., the log of a conditional probability, the first order approximation of $e^{\Phi^{\left(sp\right)}\left(n_{0}+1,n,\omega \;\right)}\;\;\;\left[1\;+\;\delta \Phi (n_{0}+1,n,\omega )\;\right]$ is not. Normalization is obtained formally by introducing the partition function:

$$Z\left[\omega_{-\infty }^{n_{0}}\;\right]\;=\sum_{\omega_{n_{0}+1}^{n}}e^{\Phi^{\left(sp\right)}\left(n_{0}+1,n,\omega \;\right)}\left[1+\delta \Phi \left(n_{0}+1,n,\omega \right)\right],$$

constrained by the past sequence $\omega_{-\infty }^{n_{0}}$, so that the quantity

$$P^{\left(1\right)}\left[\omega_{n_{0}+1}^{n}\;\left|\;\omega_{-\infty }^{n_{0}}\right.\;\right]\equiv \frac{e^{\Phi^{\left(sp\right)}\left(n_{0}+1,n,\omega \;\right)}}{Z\left[\omega_{-\infty }^{n_{0}}\;\right]}\;\left[1\;+\;\delta \Phi (n_{0}+1,n,\omega )\;\right],$$

is the first order approximation of $P\left[\omega_{n_{0}+1}^{n}\;\left|\;\omega_{-\infty }^{n_{0}}\right.\;\right]$.

Setting:

$$Z^{\left(sp\right)}\left[\omega_{-\infty }^{n_{0}}\;\right]\;=\sum_{\omega_{n_{0}+1}^{n}}e^{\Phi^{\left(sp\right)}\left(n_{0}+1,n,\omega \;\right)},$$

we have, to first order:

$$\frac{1}{Z\left[\omega_{-\infty }^{n_{0}}\;\right]}=\frac{1}{Z^{\left(sp\right)}\left[\omega_{-\infty }^{n_{0}}\;\right]}\;\left[1\;-\;\sum_{\omega_{n_{0}+1}^{n}}\;\frac{e^{\Phi^{\left(sp\right)}\left(n_{0}+1,n,\omega \;\right)}}{Z^{\left(sp\right)}\left[\omega_{-\infty }^{n_{0}}\;\right]}\;\delta \Phi \left(n_{0}+1,n,\omega \right)\;\right].$$

However, as $\Phi^{\left(sp\right)}$ is the log of a conditional probability, $Z^{\left(sp\right)}\left[\omega_{-\infty }^{n_{0}}\;\right]\;=1$. So, finally, we obtain, to first order:

$$P^{\left(1\right)}\left[\omega_{n_{0}+1}^{n}\;\left|\;\omega_{-\infty }^{n_{0}}\right.\;\right]∼P^{\left(sp\right)}\left[\omega_{n_{0}+1}^{n}\;\left|\;\omega_{-\infty }^{n_{0}}\right.\;\right]\;\left[1\;+\;\delta \Phi \left(n_{0}+1,n,\omega \right)\;-\;E^{\left(sp\right)}\left[\;\delta \Phi \left(n_{0}+1,n,.\right)\;|\;\omega_{-\infty }^{n_{0}}\right]\;\right],$$

where $E^{\left(sp\right)}\left[\;\;\right]$ denotes the expectation with respect to $\mu^{\left(sp\right)}$. We use $E^{\left(sp\right)}\left[\;\;\right]$ instead of $E_{\mu^{\left(sp\right)}}\left[\;\;\right]$ to alleviate notations.

### 3.2. Time Dependent Average of an Observable

We now consider a time-dependent observable *f* with finite range $R\equiv R_{f}$. We assume $t-t_{0}>R_{f}$. We set $R_{f}=D_{f}+1$. Setting $n=\;\left[\;t\;\right]$ we note $E_{n}\left[\;f(t,.)\;\right]\;=\int f\left(t,\omega \right)\mu \left(d\omega \right)$. Here, a note of explanation is necessary. Functions f(t,ω) are random functions, where the randomness comes from ω. So, the law of f(t,ω) is determined by the probability μ. $E_{n}\left[\;f(t,.)\;\right]$ is the average of the continuous time dependent observable f(t,ω), averaged over the discrete time spike train ω, up to the discrete time $n=\;\left[\;t\;\right]$ (by definition f(t,.) does not depend on spike events occurring at times posterior to *n*). Note that this average cannot be defined by an ergodic time average procedure as, here, the probability is non stationary (see Section 5 for a numerical implementation).

Because *f* has finite range $R_{f}$ we may write:

$$E_{n}\left[\;f(t,.)\;\right]\;=\sum_{\omega_{n-D_{f}}^{n}}f\left(t,\omega_{n-D_{f}}^{n}\;\right)\mu \left[\omega_{n-D_{f}}^{n}\right]=\sum_{\omega_{n_{0}+1}^{n}}f\left(t,\omega_{n-D_{f}}^{n}\;\right)\mu \left[\omega_{n_{0}+1}^{n}\right].$$

The last equality holds because $f\left(t,\omega \;\right)$ is independent of $\omega_{-\infty }^{n_{0}}$. Thus, using (15):

$$\begin{array}{ccc} E_{n}\left[\;f(t,.)\;\right] & = & \sum_{\omega_{n_{0}+1}^{n}}f\left(t,\omega_{n-D_{f}}^{n}\;\right)\;\int_{A_{-\infty }^{n_{0}}}\;P\left[\omega_{n_{0}+1}^{n}\;\left|\;\omega_{-\infty }^{n_{0}}\right.\;\right]\;\mu \left(d\omega \right)\\ & = & \sum_{\omega_{n_{0}+1}^{n}}f\left(t,\omega_{n-D_{f}}^{n}\;\right)\;\int_{A_{-\infty }^{n_{0}}}\;P\left[\omega_{n_{0}+1}^{n}\;\left|\;\omega_{-\infty }^{n_{0}}\right.\;\right]\;\mu^{\left(sp\right)}\left(d\omega \right), \end{array}$$

where the last equation holds because, on $F_{\leq n_{0}}$, $\mu =\mu^{\left(sp\right)}$.

Thus, replacing $P\left[\omega_{n_{0}+1}^{n}\;\left|\;\omega_{-\infty }^{n_{0}}\right.\;\right]$ by $P^{\left(1\right)}\left[\omega_{n_{0}+1}^{n}\;\left|\;\omega_{-\infty }^{n_{0}}\right.\;\right]$, we obtain, up to first order:

$$E_{n}\left[\;f(t,.)\;\right]∼\sum_{\omega_{n_{0}+1}^{n}}f\left(t,\omega_{n-D_{f}}^{n}\;\right)\;\int_{A_{-\infty }^{n_{0}}}\;P^{\left(sp\right)}\left[\omega_{n_{0}+1}^{n}\;\left|\;\omega_{-\infty }^{n_{0}}\right.\;\right]\;\mu^{\left(sp\right)}\left(d\omega \right)$$

$$+\sum_{\omega_{n_{0}+1}^{n}}f\left(t,\omega_{n-D_{f}}^{n}\;\right)\;\int_{A_{-\infty }^{n_{0}}}\;P^{\left(sp\right)}\left[\omega_{n_{0}+1}^{n}\;\left|\;\omega_{-\infty }^{n_{0}}\right.\;\right]\;\delta \Phi \left(n_{0}+1,n,\omega \right)\;\mu^{\left(sp\right)}\left(d\omega \right)$$

$$-\sum_{\omega_{n_{0}+1}^{n}}f\left(t,\omega_{n-D_{f}}^{n}\;\right)\;\int_{A_{-\infty }^{n_{0}}}\;P^{\left(sp\right)}\left[\omega_{n_{0}+1}^{n}\;\left|\;\omega_{-\infty }^{n_{0}}\right.\;\right]\;E^{\left(sp\right)}\left[\;\delta \Phi \left(n_{0}+1,n,.\right)\;|\;\omega_{-\infty }^{n_{0}}\right]\;\mu^{\left(sp\right)}\left(d\omega \right).$$

The first term is $E^{\left(sp\right)}\left[\;f(t,.)\;\right]$ from (15). The second term is:

$$\sum_{\omega_{n_{0}+1}^{n}}\int_{A_{-\infty }^{n_{0}}}\;f\left(t,\omega \;\right)\;\;\delta \Phi \left(n_{0}+1,n,\omega \right)\;P\left[\omega_{n_{0}+1}^{n}\;\left|\;\omega_{-\infty }^{n_{0}}\right.\;\right]\;\mu^{\left(sp\right)}\left(d\omega \right)=E^{\left(sp\right)}\left[\;f\left(t,.\right)\;\delta \Phi \left(n_{0}+1,n,.\right)\;\right]$$

from the consistency property (10), and because by assumption ($n-n_{0}>R_{f}$), $f\left(t,\omega \;\right)$ does not depend on $\omega_{-\infty }^{n_{0}}$.

For the third term:

$$\sum_{\omega_{n_{0}+1}^{n}}\;f\left(t,\omega_{n-D_{f}}^{n}\;\right)\;\int_{A_{-\infty }^{n_{0}}}E^{\left(sp\right)}\left[\;\delta \Phi \left(n_{0}+1,n,\omega \right)\;|\;\omega_{-\infty }^{n_{0}}\right]\;P^{\left(sp\right)}\left[\omega_{n_{0}+1}^{n}\;\left|\;\omega_{-\infty }^{n_{0}}\right.\;\right]\;\;\mu^{\left(sp\right)}\left(d\omega \right)$$

$$=E^{\left(sp\right)}\left[\;f\left(t,.\right)\;E^{\left(sp\right)}\left[\;\delta \Phi \left(n_{0}+1,n,\omega \right)\;|\;\omega_{-\infty }^{n_{0}}\right]\;\right].$$

However, by assumption $f(t,\omega_{n-D_{f}}^{n})$ does not depend on $\omega_{-\infty }^{n_{0}}$ ($n-n_{0}>D_{f}$), whereas by definition of the conditional expectation $E^{\left(sp\right)}\left[\;\delta \Phi \left(n_{0}+1,n,\omega \right)\;|\;\omega_{-\infty }^{n_{0}}\right]$ is the projection on the sigma-algebra $F_{\leq n_{0}}$. As a consequence, we have:

$$\begin{array}{ccc} E^{\left(sp\right)}\left[\;f\left(t,.\right)\;E^{\left(sp\right)}\left[\;\delta \Phi \left(n_{0}+1,n,.\right)\;|\;\omega_{-\infty }^{n_{0}}\right]\;\right] & = & E^{\left(sp\right)}\left[\;f(t,.)\;\right]\;E^{\left(sp\right)}\left[\;E^{\left(sp\right)}\left[\;\delta \Phi \left(n_{0}+1,n,.\right)\;|\;\omega_{-\infty }^{n_{0}}\right]\;\right]\\ & = & E^{\left(sp\right)}\left[\;f(t,.)\;\right]\;E^{\left(sp\right)}\left[\;\delta \Phi (n_{0}+1,n,.)\;\right]. \end{array}$$

Summing up, we have, using (13):

$$E_{n}\left[\;f(t,.)\;\right]\;=E^{\left(sp\right)}\left[\;f(t,.)\;\right]\;+\sum_{r=n_{0}+1}^{n=\left[\;t\;\right]}\left(E^{\left(sp\right)}\left[\;f(t,.)\;\delta \phi (r,.)\;\right]-E^{\left(sp\right)}\left[\;f(t,.)\;\right]\;E^{\left(sp\right)}\left[\;\delta \phi (r,.)\;\right]\;\right),$$

Using the correlation function:

$$C^{\left(sp\right)}\left[f\left(t,.\right),g\left(t^{\prime },.\right)\;\right]\;\overset{\mathrm{def}}{=}E^{\left(sp\right)}\left[\;f\left(t,.\right)g\left(t^{\prime },.\right)\;\right]\;-\;E^{\left(sp\right)}\left[\;f(t,.)\;\right]E^{\left(sp\right)}\left[\;g(t^{\prime },.)\;\right]$$

we obtain

(16) $$\delta^{\left(1\right)}\left[f(t,.)\;\right]\;=\sum_{r=n_{0}+1}^{n=\left[\;t\;\right]}C^{\left(sp\right)}\left[f(t,.),\delta \phi (r,.)\;\right].$$

This equation expresses that the time-dependent variation in the average of an observable *f* is expressed, to the first order, as a time series of correlation functions, between *f* and the time-dependent variation of the normalized potential, computed with respect to the equilibrium distribution. This is our main result.

It is similar to the fluctuation-dissipation theorem in statistical physics [32,33]. Here, it holds for Gibbs distributions with infinite range potential ϕ(t,ω). A crucial point is the convergence of the series when the initial time of perturbation, $n_{0}$ tends to −∞. This holds if correlations $C^{\left(sp\right)}\left[f(t,.),\delta \phi (r,.)\;\right]$ decay sufficiently fast, typically, exponentially. We consider only this case in this article.

One of the advantages of this relationship is that averages are taken with respect to $\mu^{\left(sp\right)}$. In the case of experimental data, these averages can be approximated by empirical averages on spontaneous activity. The monomial decomposition of (16) can be found in the Appendix B.

## 4. An Example: Linear Response in a Conductance Based Integrate and Fire Model

### 4.1. Discrete Time Integrate and Fire Network Model

The Leaky Integrate and Fire model (LIF) consider a point neuron *k* (without spatial extension), with membrane potential $V_{k}$, membrane capacity $C_{k}$, resistance *R*, submitted to a current $I_{k}\left(t\right)$. Call θ the spiking threshold. The sub-threshold dynamics is:

(17) $$C_{k}\;\frac{dV_{k}}{dt}+\;\frac{1}{R}V_{k}=I_{k}\left(t\right),\;\mathrm{if}\;V_{k}\left(t\right)<\theta .$$

If there is a time $t_{k}$ such that the membrane potential of neuron *k* reaches the firing threshold, $V_{k}\left(t_{k}\right)\geq \theta$, the neuron *k* fires an action potential, i.e., it emits a spike and the membrane potential of neuron *k* is reset to a fixed reset value $V_{res}$ instantaneously. The neuron’s membrane potential remains at this value during a time denoted by Δ called “refractory period”, i.e., $V_{k}\left(t^{\prime }\right)=V_{res},\;t^{\prime }\in \left[t_{k},t_{k}+\Delta \right]$.

To illustrate our results we use a “simple” model corresponding to a discrete-time LIF network [34]. The main reason to choose this model is to facilitate numerical simulations as handling continuous-time dynamics with spikes event, although manageable mathematically [57] is difficult to handle numerically [58]. To further simplify the analysis we fixing the sampling time dt=1, the capacitance $C_{k}=1$, τ=RC, γ=1−1/τ,∈[0,1[ is called the“leak rate”. The evolution of the membrane potential $V_{k}$ is ruled by the following sub-threshold equation:

$$V_{k}\left(n+1\right)=\gamma \;V_{k}\left(n\right)+I_{k}\left(t\right).$$

Note that by setting $C_{k}=1$ we are not considering units of measurements anymore.

We now explicitly consider an interconnected network of neurons. The network of synaptic connectivity is represented by a matrix of components $W_{kj}$ which can be positive or negative to characterize inhibition or excitation respectively. We consider random fluctuations by adding a standard Gaussian additive noise $\xi_{k}\left(n\right)$ controlled by the amplitude $\sigma_{B}$. We also consider a constant stimulus $I_{0}$ and a time dependent stimulus $S_{k}\left(t\right)$. Thus $I_{k}\left(t\right)=\sum_{j}W_{kj}\omega_{j}\left(n\right)+I_{0}+S_{k}\left(t\right)+\sigma_{B}\xi_{k}\left(n\right)$, and thus our equation finally reads:

(18) $$V_{k}\left(n+1\right)=\gamma \;V_{k}\left(n\right)+\sum_{j}W_{kj}\omega_{j}\left(n\right)+I_{0}+S_{k}\left(t\right)+\sigma_{B}\xi_{k}\left(n\right),\;\mathrm{if}\;V_{k}\left(n\right)<\theta .$$

Note that γ, the decay term, is related to the leak characteristic time (18) by:

$$\tau =\frac{1}{1-\gamma }.$$

The condition γ<1 define the exponential decay in the spike history dependence via the characteristic time:

(19) $$\tau_{\gamma }=-\left[\;\frac{1}{\log\gamma }\;\right].$$

This characteristic time can be interpreted as follows. Integrating Equation (18) up to the last time where voltage was reset in the past, $\tau_{k}\left(n,\omega \right)$ gives the following:

(20) $$V_{k}\left(n+1,\omega \right)=\sum_{j=1}^{N}W_{kj}\;\eta_{kj}\left(n,\omega \right)+I_{0}\;\frac{1-\gamma^{n+1-\tau_{k}\left(n,\omega \right)}}{1-\gamma }+\sum_{l=\tau_{k}\left(n,\omega \right)}^{n}\gamma^{n-l}S_{k}\left(l\right)+\sigma_{B}\sum_{l=\tau_{k}\left(n,\omega \right)}^{n}\gamma^{n-l}\xi_{k}\left(l\right),$$

where:

$$\eta_{kj}\left(n,\omega \right)=\sum_{l=\tau_{k}\left(n,\omega \right)}^{n}\gamma^{n-l}\;\omega_{j}\left(l\right),$$

The first term on the right-hand side of (20) corresponds to the synaptic contribution. The second corresponds to the integration of the constant term $I_{0}$, used to fix the baseline activity. The third term corresponds to the integration of a time-dependent stimulus $S_{k}\left(t\right)$ and the fourth to the integrated noise term with intensity $\sigma_{B}$.

For each n∈Z, conditionally to $\omega_{-\infty }^{n-1},V_{k}\left(n\right)$ is Gaussian random variable. It can be decomposed in the following way:

$$V_{k}\left(n,\omega \right)=V_{k}^{\left(syn\right)}\left(n,\omega \right)+V_{k}^{\left(I\right)}\left(n,\omega \right)+V_{k}^{\left(S\right)}\left(n,\omega \right)+V_{k}^{\left(noise\right)}\left(n,\omega \right)$$

We now consider the “spontaneous” voltage $V_{k}^{\left(sp\right)}\left(n,\omega \right)$ and the evoked response due to the external time dependent stimulus $V_{k}^{\left(S\right)}\left(n,\omega \right)$.

(21) $$\begin{array}{cc} V_{k}^{\left(sp\right)}\left(n,\omega \right) & =V_{k}^{\left(syn\right)}\left(n,\omega \right)+V_{k}^{\left(I\right)}\left(n,\omega \right)+V_{k}^{\left(noise\right)}\left(n,\omega \right)\\ \; & =\sum_{j=1}^{N}W_{kj}\;\eta_{kj}\left(n,\omega \right)+I_{0}\;\frac{1-\gamma^{n+1-\tau_{k}\left(n,\omega \right)}}{1-\gamma }+\sigma_{B}\sum_{l=\tau_{k}\left(n,\omega \right)}^{n}\gamma^{n-l}\xi_{k}\left(l\right) \end{array}$$

$$V_{k}^{\left(S\right)}\left(n,\omega \right)=\sum_{l=\tau_{k}\left(n,\omega \right)}^{n}\gamma^{n-l}S_{k}\left(l\right)$$

### 4.2. Transition Probabilities of the Discrete Time LIF Model

In the limit of small $\sigma_{B}$, the family of transition probabilities $P_{n}\left[\omega \left(n\right)\;\left|\;\omega_{-\infty }^{n-1}\right.\;\right]$ can be written explicitly in terms of the parameters of the spiking neuronal network model. The discrete-time LIF model is conditionally independent i.e., it factorizes over neurons once the spike history has been fixed [34]. The same property is held for the continuous-time version of this model [59]. However, a more complete version of this model also includes electric synapses [57]. In that case, the conditional independence is lost.

$$P_{n}\left[\omega \left(n\right)\;\left|\;\omega_{-\infty }^{n-1}\right.\;\right]=\prod_{k=1}^{N}P_{n}\left[\omega_{k}\left(n\right)\;\left|\;\omega_{-\infty }^{n-1}\right.\;\right],$$

where:

(22) $$P_{n}\left[\omega_{k}\left(n\right)\;\left|\;\omega_{-\infty }^{n-1}\right.\;\right]=\omega_{k}\left(n\right)\;\Pi \left(X_{k}\left(n-1,\omega \right)\;\right)\;+\;\left(1-\omega_{k}\left(n\right)\;\right)\;\left(1-\Pi \left(X_{k}\left(n-1,\omega \right)\;\right)\;\right),$$

with:

$$\Pi \left(x\right)=\frac{1}{\sqrt{2\pi }}\int_{x}^{+\infty }e^{-\frac{u^{2}}{2}}du,$$

and:

(23) $$X_{k}\left(n-1,\omega \right)\overset{\mathrm{def}}{=}\frac{\theta -V_{k}\left(n-1,\omega \right)}{\sigma_{k}\left(n-1,\omega \right)},$$

where,

$$\sigma_{k}^{2}\left(n-1,\omega \right)=\frac{1-\gamma^{2\left(n-\tau_{k}\left(n-1,\omega \right)\right)}}{1-\gamma^{2}}$$

corresponds to the variance of the noise integrated up to time n−1.

Combining (12) and (22), we obtain the normalized potential for the discrete time LIF model.

$$\phi \left(n,\omega \;\right)\;=\sum_{k=1}^{N}\phi_{k}\left(n,\omega \;\right),$$

where

(24) $$\phi_{k}\left(n,\omega \;\right)\;=\omega_{k}\left(n\right)\;\log\Pi \left(X_{k}\left(n-1,\omega \right)\;\right)\;+\;\left(1-\omega_{k}\left(n\right)\;\right)\;\log\left(1-\Pi \left(X_{k}\left(n-1,\omega \right)\;\right)\;\right),$$

which depends on all of the parameters of the network via the variable $X_{k}\left(n-1,\omega \right)$ (23).

**Remark** **1.** *The function Π(x) is a sigmoid which tends to 1 when x→−∞ and tends to 0 when x→∞. This has two consequences:*

1. *When $X_{k}\left(n-1,\omega \right)\to +\infty$ (which arises when $V_{k}^{\left(sp\right)}\left(n-1,\omega \right)\to -\infty$),$\;\;\Pi \left(X_{k}\left(n-1,\omega \right)\;\right)\to 0$) so that $\phi_{k}\left(n,\omega \;\right)\to -\infty$. This expresses that the probability of having a spike at time n when $X_{k}\left(n-1,\omega \right)$ becomes large (neuron strongly hyper-polarized) tends to 0. The same argument holds mutatis mutandis for the limit $X_{k}\left(n-1,\omega \right)\to -\infty$.*
2. *When $|X_{k}\left(n-1,\omega \right)|$ is large (neuron either strongly hyper-polarized or strongly depolarized) the effect of a variation of the membrane potential on the firing probability is negligible. Thus, we study the effect of a perturbation in bounded range for $X_{k}\left(n-1,\omega \right)$:*
  (25) $$0<\epsilon <\Pi \left(X_{k}\left(n-1,\omega \right)\;\right)<1-\epsilon <1,$$
*uniformly in n,ω. This is ensured by natural assumptions on synaptic weights and on $\sigma_{B}$, the mean-square deviation of the noise, which has to be bounded away from 0.*

### 4.3. Expansion of the Normalized Potential

The normalized potential of the Discrete time LIF model can be separated into: (i) a “spontaneous” part $\phi^{\left(sp\right)}\left(\omega \right)$, which before time $t_{0}$ is independent of the stimuli and time and; (ii) a “perturbation” part δϕ(n,ω) depending on a time-dependent stimuli, which is non-zero from time $t_{0}$. Mathematically this is achieved by adding an extra term to the spontaneous potential after time $t_{0}$.

$$\phi \left(n,\omega \right)=\;\left\{\begin{array}{ccc} \phi^{\left(sp\right)}\left(\omega \right)\; & \mathrm{if} & n<\;\left[\;t_{0}\;\right];\\ \phi^{\left(sp\right)}\left(\omega \right)+\delta \phi \left(n,\omega \right)\; & \mathrm{if} & n\geq \;\left[\;t_{0}\;\right]\;. \end{array}\right.$$

Note that, at this stage, this is just a definition of $\delta \phi \left(n,\omega \right)=\phi \left(n,\omega \right)-\phi^{\left(sp\right)}\left(\omega \right)$.

From Section 3 this perturbation induces a time-dependent variations on the average of an observable *f*:

$$\mu_{n}\left[f\left(n,\cdot \right)\right]=E^{\left(sp\right)}\left[\;f(n,\cdot )\;\right]+\delta^{\left(1\right)}\left[f(n,\cdot )\;\right],$$

If $n\leq t_{0},\;\delta^{\left(1\right)}\left[f(n,\cdot )\;\right]\;=0,\forall f\left(n,\cdot \right)$ as $\mu_{n}=\mu^{\left(sp\right)}$. Thus, the term $E^{\left(sp\right)}\left[\;f(n,\cdot )\;\right]$ refers to an average with respect to the unperturbed system and $\delta^{\left(1\right)}\left[f(n,\cdot )\;\right]$. As we show, the variation $\delta^{\left(1\right)}$ can be explicitly written in terms of the variation on the normalized potential induced by the introduction of the stimulus.

Note that if the external stimuli are switched on at time $t_{0}$, spike statistics are still constrained by the previous spontaneous activity, since transition probabilities have memory. This effect is especially salient in the discrete-time LIF model which has an unbounded memory.

We rewrite (23) in the form:

$$X_{k}\left(n-1,\omega \right)=X_{k}^{\left(sp\right)}\left(n-1,\omega \right)\;+\;\delta X_{k}\left(n-1,\omega \right),$$

where

$$X_{k}^{\left(sp\right)}\left(n-1,\omega \right)=\frac{\theta -V_{k}^{\left(sp\right)}\left(n-1,\omega \right)}{\sigma_{k}\left(n-1,\omega \right)}$$

is independent of the stimulus, and:

$$\delta X_{k}\left(n-1,\omega \right)=-\frac{V_{k}^{\left(S\right)}\left(n-1,\omega \right)}{\sigma_{k}\left(n-1,\omega \right)}=-\frac{1}{\sigma_{k}\left(n-1,\omega \right)}\sum_{l=\tau_{k}\left(n-1,\omega \right)}^{n-1}\gamma^{n-1-l}S_{k}\left(l\right),$$

where the last equality holds because S(n)=0 for $n<t_{0}$.

In the next computation we write $X_{k}^{\left(sp\right)},\delta X_{k}$ instead of $X_{k}^{\left(sp\right)}\left(n-1,\omega \right),\delta X_{k}\left(n-1,\omega \right)$ to alleviate notations. We make a series expansion of $\phi_{k}\left(n,\omega \;\right)$ at $X_{k}^{\left(sp\right)}$, under the conditions of (25). We have:

$$\begin{array}{c} \log\Pi \left(X_{k}^{\left(sp\right)}+\delta X_{k}\right)=\log\Pi \left(X_{k}^{\left(sp\right)}\right)+\sum_{u=1}^{+\infty }\frac{a^{\left(u\right)}\left(X_{k}^{\left(sp\right)}\right)}{u!}\;{\left(\delta X_{k}\;\right)}^{u},\\ \log\left(1-\Pi (X_{k}^{\left(sp\right)}+\delta X_{k})\;\right)=\log\left(1-\Pi (X_{k}^{\left(sp\right)})\;\right)+\sum_{u=1}^{+\infty }\frac{b^{\left(u\right)}\left(X_{k}^{\left(sp\right)}\right)}{u!}\;{\left(\delta X_{k}\;\right)}^{u} \end{array}$$

where $a^{\left(u\right)}$ and $b^{\left(u\right)}$ are the *u*-th derivative of logΠ(x) and log(1−Π(x)). In particular:

$$a^{\left(1\right)}\left(x\right)=\frac{\Pi^{\prime }\left(x\right)}{\Pi (x)};\;\;b^{\left(1\right)}\left(x\right)=-\frac{\Pi^{\prime }\left(x\right)}{1-\Pi (x)}.$$

Therefore,

$$\delta \phi_{k}\left(n,\omega \;\right)\;=\sum_{u=1}^{+\infty }\delta \phi_{k}^{\left(u\right)}\left(n,\omega \;\right),$$

where:

$$\delta \phi_{k}^{\left(u\right)}\left(n,\omega \;\right)\;=H_{k}^{\left(u\right)}\left(n,\omega \right)\;{\left[\delta X_{k}\left(n-1,\omega \right)\;\right]}^{u},$$

with:

(26) $$H_{k}^{\left(u\right)}\left(n,\omega \right)=\frac{1}{u!}\left[\omega_{k}\left(n\right)\;a^{\left(u\right)}\;\left(X_{k}^{\left(sp\right)}\left(n-1,\omega \right)\;\right)\;+\left(1-\omega_{k}\left(n\right)\;\right)b^{\left(u\right)}\;\left(X_{k}^{\left(sp\right)}\left(n-1,\omega \right)\right)\;\right].$$

This expansion holds for any value of $X_{k}^{\left(sp\right)}\left(n-1,\omega \right)$. However, when this quantity becomes large in absolute value, one has to consider more and more terms in the expansion to approach sufficiently well the function $\delta \phi_{k}\left(n,\omega \;\right)$. This is a well-known property of the function Π (which can be written in terms of the error function): the Taylor expansion converges very slowly near infinity and other expansions are more efficient (e.g., Bürmann series [60]). Here, we consider the effect of a perturbation in a range where the function Π does not saturate. In addition, we restrict ourselves to cases where the first order of the Taylor expansion is sufficient to characterize the response. This is ensured by conditions of the form (the same holds mutatis mutandis for *b*):

$$|\delta X_{k}|\ll {\left(u!\;\right)}^{\frac{1}{u-1}}{\left|\frac{a^{\left(1\right)}\left(X_{k}^{\left(sp\right)}\;\right)}{a^{\left(u\right)}\left(X_{k}^{\left(sp\right)}\;\right)}\right|}^{\frac{1}{u-1}};\;u>1.$$

Applied to the second order this gives a condition:

(27) $$|\delta X_{k}\left(n-1,\omega \right)|\ll \frac{2}{|X_{k}^{\left(sp\right)}\left(n-1,\omega \right)+a^{\left(1\right)}\left(X_{k}^{\left(sp\right)}\left(n-1,\omega \right)\;\right)|},$$

which becomes more and more restrictive as one gets away from $X_{k}^{\left(sp\right)}\left(n-1,\omega \right)=0$ i.e., away from the linear region of the sigmoid Π. Note that the condition $X_{k}^{\left(sp\right)}\left(n-1,\omega \right)∼0$ corresponds to a voltage close to the firing threshold. We insist that this constraint is not a limitation of our approach, but instead, a limitation of linear response applied in neuronal systems where the response of a neuron is characterized by a saturating function. Away from the linear part of the sigmoid, nonlinear effects dominate the response. In the numerical simulations, we use the mean-field approximation where we replace $\tau_{k}\left(r-1,.\right)$ by its average value $\mu \left[\tau_{k}\left(r-1,.\right)\;\right]\;=r-1-\frac{1}{\nu_{k}}$ where the inverse $\frac{1}{\nu_{k}}$ of the average firing rate $\nu_{k}$ of neuron *k* is the mean time between two spikes.

Under these conditions we obtain:

$$\delta \phi_{k}^{\left(1\right)}\left(r,\omega \;\right)\;=-\frac{H_{k}^{\left(1\right)}\left(r,\omega \right)}{\sigma_{k}\left(r-1,\omega \right)}\;\sum_{l=r-1-\frac{1}{\nu_{k}}}^{r-1}\gamma^{r-1-l}\;S_{k}\left(l\right)$$

where we have replaced *n* by *r* in view of (16).

The function $\delta \phi_{k}^{\left(1\right)}\left(r,\omega \;\right)$ is the first order variation of normalized potential when neurons are submitted to a weak time dependent stimulus, under the approximation (24). There are two contributions. The integral includes the effect of the stimulus on the dynamics flow; the term $H_{k}^{\left(1\right)}\left(r,\omega \right)$, given by Equation (26), contains the effect of the network via the terms $a^{\left(1\right)}\left(\frac{\theta -V_{k}^{\left(sp\right)}\left(r-1,\omega \right)}{\sigma_{k}\left(n-1,\omega \right)}\;\right)$, $b^{\left(1\right)}\left(\frac{\theta -V_{k}^{\left(sp\right)}\left(r-1,\omega \right)}{\sigma_{k}\left(n-1,\omega \right)}\;\right)$ where $V_{k}^{\left(sp\right)}\left(r-1,\omega \right)$ is given by Equation (21). Note that the dependence on synaptic weights is non-linear because $a^{\left(1\right)}$ and $b^{\left(1\right)}$ are non-linear.

In order to study the linear response theory in this model, one has to properly handle numerically:

(1) The spike train statistics, especially finding a numerical way to perform a suitable averaging, not only for the spontaneous probability measure where time-ergodic average can be used but also for the non-stationary response where ergodicity does not take place;
(2) The long memory tail in the dynamics;
(3) Find an illustrative example with a good range of parameters showing convincing, original, non-trivial results while avoiding prohibitive computational times.

It is not evident to us that classical spiking neural network simulators such as BRIAN [61], although quite efficient, could easily handle (1) in conjunction with (2). The model presented below, has been studied both from the mathematical side and the numerical side [8,34]. Additionally, we have designed a simulation tool, PRANAS, devoted to the analysis of population spike train statistics and allowing to properly handle Gibbs distributions from numerical simulations or experimental data, with a specific module dedicated to this model [62]. This software is freely downloadable at https://team.inria.fr/biovision/pranas-software/ on simple demand. The linear response C++ codes and instructions required to reproduce these numerical results can be found at https://github.com/brincolab/Linear-response.

### 4.4. Linear Response

We apply our main result (16) to the potential of the discrete-time model (24). Under the approximations made in the previous section, we examine two forms of linear response proposed in the paper.

1. The first-order expansion of the potential, (16). In the present context it becomes:
  $$\delta \mu^{\left(1\right)}\left[f(n)\;\right]\;∼-\;\sum_{k=1}^{N}\;\sum_{r=-\infty }^{n}\sum_{l=r-1-\frac{1}{\nu_{k}}}^{r-1}\gamma^{r-1-l}C^{\left(sp\right)}\left[f(n,\cdot ),\zeta (r-1,.)\;\right]\;S_{k}\left(l\right),$$
  where we have set $\zeta_{k}\left(r-1,\omega \right)=\frac{H_{k}^{\left(1\right)}\left(r,\omega \right)}{\sigma_{k}\left(r-1,\omega \right)}$. In the numerical simulations we use a Markovian approximation with memory depth *D* such that the sum $\sum_{r=-\infty }^{n}$ is replaced by $\sum_{r=n-D}^{n}$. *D* is typically determined by exponential decay of the terms $\gamma^{l-r+1}C^{\left(sp\right)}\left[f\left(n,\cdot \right),\zeta_{k}\left(r-1,.\right)\;\right]$, which is controlled, on one hand, by γ (with a characteristic time $\tau_{\gamma }=-\left[\;\frac{1}{\log\gamma }\;\right]$), and the correlation $C^{\left(sp\right)}\left[f\left(n,\cdot \right),\zeta_{k}\left(r-1,.\right)\;\right]$, which is controlled by the spectral gap in the Perron–Frobenius matrix. In this approximation we have:
  $$\delta \mu^{\left(1\right)}\left[f(n)\;\right]\;∼-\;\sum_{k=1}^{N}\;\sum_{r=n-D}^{n}\sum_{l=r-1-\frac{1}{\nu_{k}}}^{r-1}\gamma^{r-1-l}C^{\left(sp\right)}\left[f\left(n,\cdot \right),\zeta_{k}\left(r-1,.\right)\;\right]\;S_{k}\left(l\right).$$
  Using the stationarity of $\mu^{\left(sp\right)}$ we have $C^{\left(sp\right)}\left[f(n,\cdot ),\zeta (r-1,.)\;\right]\;=C^{\left(sp\right)}\left[f(n-r+1,\cdot ),\;\;\right]\;\zeta_{k}\left(0,.\right)$ where r∈[n−D,n] so that m=n−r+1∈[1,D+1]. Then, introducing the N×D matrix $K^{\left(1\right)}$ with entries:
  $$K_{k,m}^{\left(1\right)}=C^{\left(sp\right)}\left[f\left(m,\cdot \right),\zeta_{k}\left(0,.\right)\;\right],$$
  we may write $\delta \mu^{\left(1\right)}\left[f(n)\;\right]$ in the form:
  (28) $$\delta \mu^{\left(1\right)}\left[f(n)\;\right]\;∼-\;\sum_{k=1}^{N}\;\sum_{m=1}^{D+1}\sum_{l=0}^{\frac{1}{\nu_{k}}}\;\gamma^{l}\;K_{km}^{\left(1\right)}\;S_{k}\left(n-m-l\right).$$
2. The first order Hammersley–Clifford expansion of $H_{k}^{\left(1\right)}$ which corresponds to expand $\zeta_{k}\left(0,\omega \right)$ to the lowest order in the Hammersley–Clifford expansion, $\zeta_{k}\left(0,\omega \right)∼\gamma_{k}^{\left(1\right)}\omega_{k}\left(0\right)$ with:
  $$\gamma_{k}^{\left(1\right)}=a^{\left(1\right)}\left(\theta_{L,k}\right)-b^{\left(1\right)}\left(\theta_{L,k}\right),$$
  with $a^{\left(1\right)}\left(x\right)=\frac{\Pi^{\prime }\left(x\right)}{\Pi (x)}$, $b^{\left(1\right)}\left(x\right)=-\frac{\Pi^{\prime }\left(x\right)}{1-\Pi (x)}$. This gives an approximation similar to the fluctuation-dissipation theorem where the linear response is a sum of pairwise correlation functions. In the discrete time model it reads:
  (29) $$\delta \mu^{\left(HC_{1}\right)}\left[f(n)\;\right]\;∼-\frac{2\;\sqrt{1-\gamma }}{\sigma_{B}}\;\gamma^{\left(1\right)}\;\;\sum_{k=1}^{N}\;\sum_{m=1}^{D+1}\sum_{l=0}^{\frac{1}{\nu_{k}}}\gamma^{l}\;K_{km}^{\left(HC_{1}\right)}\;S_{k}\left(n-m-l\right).$$
  where:
  $$K_{k,m}^{\left(HC_{1}\right)}=C^{\left(sp\right)}\left[f\left(m,\cdot \right),\omega_{k}\left(0,.\right)\;\right].$$
  We have used the superscript “$HC_{1}$” to refer to the lowest-order Hammersley–Clifford approximation from (28). The interest of this approximation (and more generally, of the Hammersley–Clifford expansion is that it can be obtained without knowing explicitly the potential ϕ (by a mere fit of the coefficients $\gamma_{k}^{\left(1\right)}$). We expect however (28) to give a better approximation of the linear response than (29). Note that the effective threshold corresponds to $\theta_{L,k}=\frac{\theta -\frac{I_{0}}{1-\gamma }}{\sqrt{\frac{1}{2(1-\gamma )}}\;\sigma_{B}},$ is independent of *k* in this case. This explains why $\gamma^{\left(1\right)}$ gets out of the sum and lost its index *k* in (29).

## 5. Numerical Simulations

### 5.1. Averaging Method

We need to compute numerically averages with respect to the invariant probability $\mu^{\left(sp\right)}$. As $\mu^{\left(sp\right)}$ is ergodic it is in principle possible to get them by time averaging. However, we also want to compute averages in the presence of a stimulus, where ergodicity does not take place. In addition, a notation like $E^{\left(sp\right)}\left[\;f(n,\cdot )\;g(r,\cdot )\;\right]$ appearing all over the paper involves a subtlety: we are computing the average of time-dependent quantities (via the first argument in f(n,·),g(r,·)) with respect to an invariant probability on the second argument, ω. The mathematical meaning is the following:

$$E^{\left(sp\right)}\left[\;f(n,\cdot )\;g(r,\cdot )\;\right]\;=\int_{\Omega }f\left(n,\omega \right)\;g\left(r,\omega \right)\mu^{\left(sp\right)}\left(d\omega \right).$$

We numerically compute such quantities by generating *M* spike trains, denoted $\omega^{\left(m\right)}$, m=1…M, of length *T*, with the spontaneous dynamics, thus distributed according to $\mu^{\left(sp\right)}$. Then:

(30) $$\int_{\Omega }f\left(n,\omega \right)\;g\left(r,\omega \right)d\mu^{\left(sp\right)}\left[\omega \;\right]∼\frac{1}{M}\;\sum_{m=1}^{M}f\left(n,\omega^{\left(m\right)}\right)\;g\left(r,\omega^{\left(m\right)}\right).$$

The equality holds in the limit M→∞. Here, we chose *M* = 10,000 or *M* = 100,000. Fluctuations about the mean are ruled by the central limit theorem, so they decrease like $\frac{1}{\sqrt{M}}$ with a proportionality factor depending on the observables *f* and *g*.

### 5.2. Results

**Figure 2:**
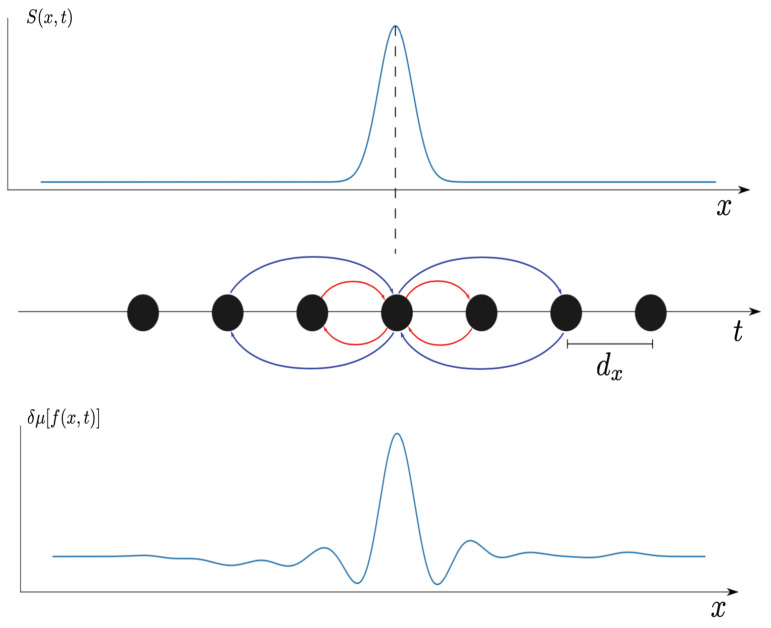
Connectivity pattern of the model. Neurons (black filled circles) are located into a one-dimensional lattice with spacing $d_{x}$. They are connected to nearest neighbors through excitatory connections (red) with weight $w^{+}$ and to second nearest neighbors through inhibitory connections (blue) with weight $w^{-}$. These neurons are submitted to a space-time dependent stimulus S(x,t) (top line, blue trace) traveling at speed *v* from left to right. This modifies the average activity $\mu \left[f(x,t)\;\right]$ by a variation $\delta \mu \left[f(x,t)\;\right]$ (bottom trace).

We consider a one-dimensional lattice of N=30 neurons, separated by a lattice spacing $d_{x}$, with null boundary conditions. The connectivity is depicted in Figure 2. Each neuron excites its neighbours with a weight $W_{k\pm 1,k}=w^{+}>0$ and inhibits its second neighbours with a weight $W_{k\pm 2,k}=w^{-}<0$. We compare the spontaneous activity (with a noise term and a constant term $I_{0}$ to fix the baseline activity, as in (18)) to the dynamics in the presence of Gaussian pulse, with width Δ, propagating at speed *v* from left to right:

(31) $$S\left(x,t\right)=\frac{A}{\sqrt{2\pi }\;\Delta }e^{-\frac{1}{2}\;\frac{{\left(x-v\;t\;\right)}^{2}}{2\;\Delta^{2}}},$$

where x,t are continuous space-time variables. The neuron *k* is located at $x_{k}=k\;\delta$ and time is updated at each t=nb where *b* is a time bin. In the simulations $d_{x}=1\;\mathrm{mm},\;v=2\;\mathrm{mm}/s,\;b=10\;\mathrm{ms},\;\Delta =1\;\mathrm{mm}$. The term *A* represent the amplitude and is variable to show the validity of the linear response theory as the amplitude of the stimulus increases. We fix θ=1, γ=0.6, corresponding to a characteristic decay time (19) $\tau_{\gamma }=-\left[\;\frac{1}{\log\gamma }\;\right]∼2$. The memory *D* appearing in the summations (28), (29) was taken to be D=10 from the study of correlation decay rate. This is a good compromise between the convergence of these sums and the computational time.

**Figure 3:**
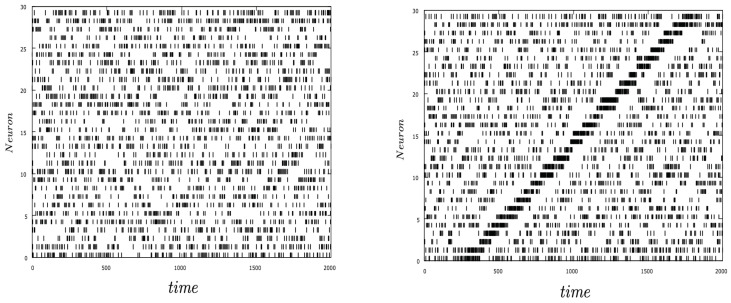
Spiking activity of the network. Left panel shows the spontaneous activity and right panel in the presence of a moving stimulus (31).

The constant stimulus $I_{0}$ is fixed to have a good baseline activity. We consider the activity without and with stimulus with moderate excitatory connectivity and strong inhibitory connectivity ($w^{+}=0.2$, $w^{-}=2$). In Figure 3 we show the spike network activity in spontaneous activity (left) and in the presence of a moving stimulus (right). The strong inhibition is particularly visible in the presence of the stimulus.

### 5.3. Linear Response for Firing Rates

**Figure 4:**
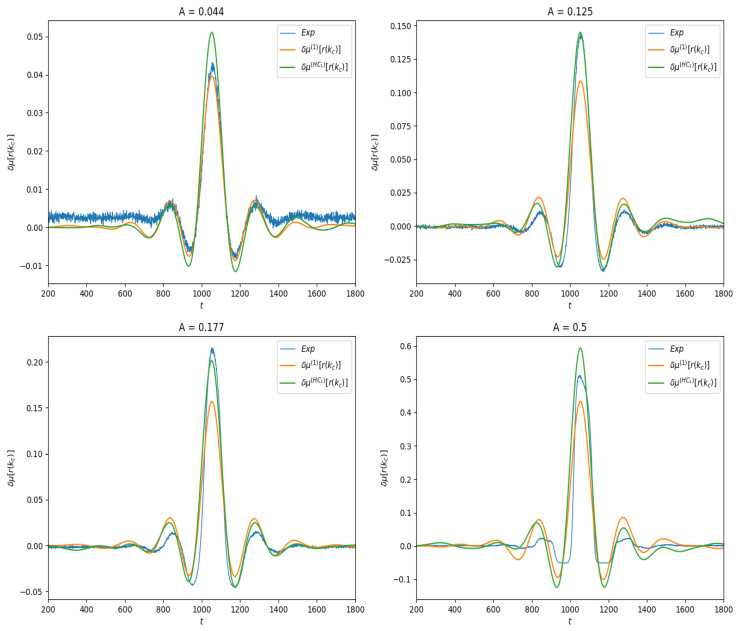
Linear response of $f\left(\omega ,n\right)=\omega_{k_{c}}\left(n\right)$ for different values of stimulus amplitude *A*. Blue curve: Empirical average trace computed from (30). Orange: Linear response computed from Equation (28). Green: Linear response computed from Equation (29).

We first present the results of linear response for the observable $f\left(n,\omega \right)=\omega_{k_{c}}\left(n\right)$, where $k_{c}=\frac{N}{2}$ is the index of the neuron located at the center of the lattice. Thus, $\mu \left[f(n,\omega )\;\right]\;\equiv r\left(k_{c},t\right)$, the firing rate of this neuron as a function of time. In spontaneous activity it is a constant; under stimulation it depends on time. In Figure 4 we show the effect of the stimulus on the average value of this observable for different amplitudes values. One observes the combined effect of the stimulus and of the connectivity.

**Figure 5:**
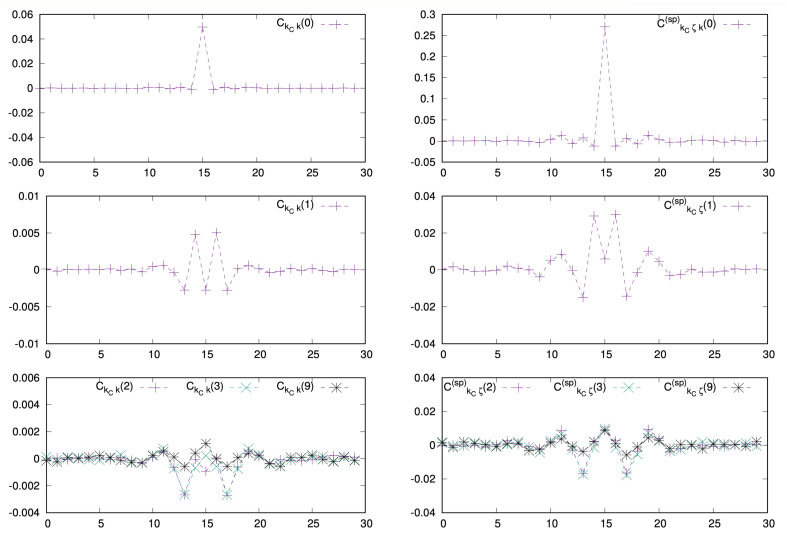
Correlation functions corresponding to the firing rate of the neuron $k_{c}=\frac{N}{2}$ as a function of the neuron index *k* (abscissa), for different values of the time delay *m*. (**Left**) correlations with stimulus. (**Right**) correlations in the spontaneous regime. Top. m=0, middle m=1, bottom m=2,3,9.

We next studied how correlation functions in spontaneous activity depend on space and time. One observes that they decay relatively fast with the time delay of *m* (Figure 5). In addition, they are multiplied by $\gamma^{m}$ in (28), (29). Therefore the contribution to the linear response series decay exponentially fast and the series can be truncated to low order. Here we took a maximal order D=10.

Finally, as shown in Figure 4, we compute the linear response $\delta \mu^{\left(1\right)}\left[f(t)\;\right],\delta \mu^{\left(HC_{1}\right)}\left[f(t)\;\right]$ and compare them to the response obtained by empirical averages.

### 5.4. Linear Response for Higher Order Observables

Here, we consider the pairwise observable $f\left(\omega ,n\right)=\omega_{k_{c}-2}\left(n-3\right)\omega_{k_{c}}\left(n\right)$ where $k_{c}=\frac{N}{2}$. This is an example of an observable with a time delay. Neurons $k_{c}-2$ and $k_{c}$ mutually inhibit each other so we expect that the state of neuron $k_{c}-2$ before *n* impact the state of neuron $k_{c}$ at time *n*. However, the correlation between those states depends as well on the state of the other neurons.

**Figure 6:**
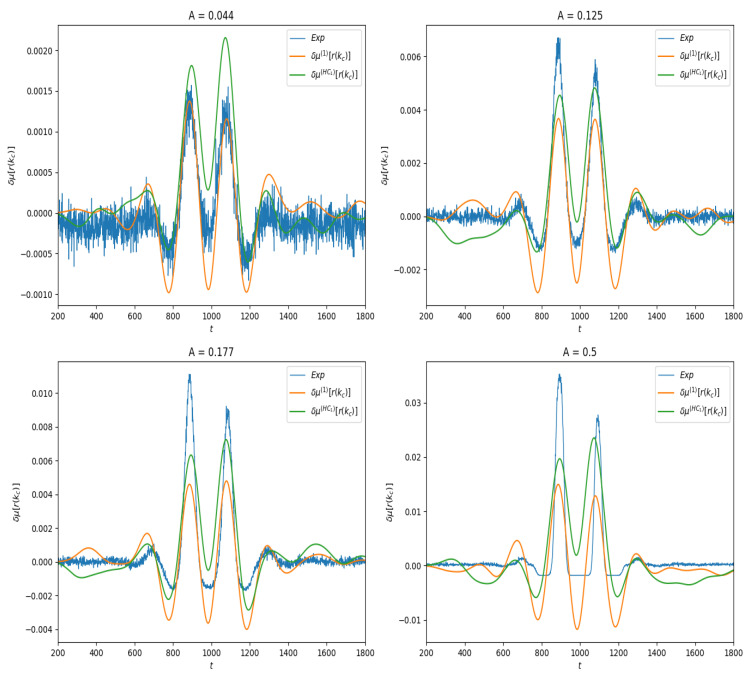
Linear response of the observable $f\left(n,\omega \right)=\omega_{k_{c}-2}\left(n-3\right)\omega_{k_{c}}\left(n\right)$. Here, we consider the same amplitudes and as Figure 4.

Similarly to the previous section we have plotted in Figure 6 the empirical estimation of the linear response under the two approximations (28), (29).

### 5.5. Validity of the Linear Response

**Figure 7:**
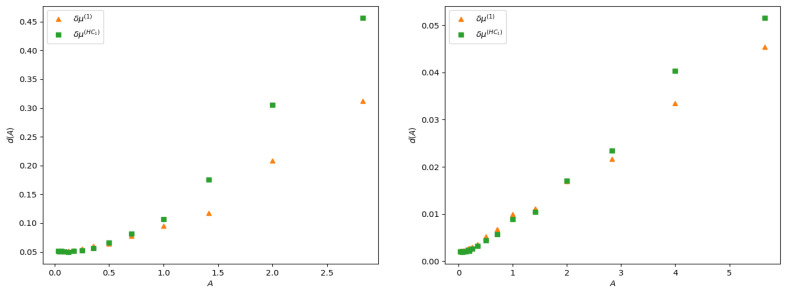
$d^{2}$ distance (32) between the curves $\delta \mu^{\left(1\right)}\left[f(n)\;\right]$, $\delta \mu^{\left(HC_{1}\right)}\left[f(n)\;\right]$ and the empirical curve, as a function of the stimulus amplitude *A*. The left panel shows the distance between rate curves (Section 5.3) and right panel distance between pairwise with delay (Section 5.4).

The linear response is expected to hold when the stimulus amplitude is weak. What does that mean? A mathematical answer is given by Equation (27) although remaining at a rather abstract level. Here, we compute the distance:

(32) $$d^{2}\left(\delta \mu^{\left(th\right)}\left[f(n)\;\right],\delta \mu^{\left(exp\right)}\left[f(n)\;\right]\right)=\sum_{n=1}^{T}{\left(\delta \mu^{\left(th\right)}\left[f(n)\;\right]-\delta \mu^{\left(exp\right)}\left[f(n)\;\right]\;\right)}^{2},$$

between the theoretical curves $\delta \mu^{\left(th\right)}\left[f(n)\;\right]$ and the experimental curve $\delta \mu^{\left(exp\right)}\left[f(n)\;\right]$, as a function of the stimulus amplitude *A*, in the two examples of observable investigated here. Note that distance here is not normalized: it does not take into account the amplitude of the response. This explains why the distance is larger in the case of firing rates than in the delayed pairwise case, as in the latter the norm of the curve is quite smaller. The result is presented in Figure 7. As expected, the error in both cases increases with the amplitude of the stimulus, and it increases slower for (28) than for the lowest order Hammersley–Clifford expansion (29). It is interesting to see how the curves differ when *A* increases (see Figure 4 and Figure 6). The empirical average curves clearly exhibit a non-linear saturation (e.g., firing rate cannot exceed 1) that is not reproduced by the linear response theory. This is further commented on in the discussion section.

### 5.6. Comments on Numerical Results

With these simulations, we have been able to illustrate our results. We are able to predict the time variation of an observable under a non-stationary stimulation, from the mere knowledge of the stimulus and the spontaneous statistics. In particular, our results hold for observables with time delays, considerably enlarging the scope of linear response theories in neuronal networks. The comparison with classical fluctuation theory also emphasizes the role played by higher-order terms. Linear response requires that the stimulus has a weak enough amplitude. We see very well this effect numerically. As the amplitude *A* of the stimulus increases, we check the increasing discrepancy between the empirical averages and the prediction.

## 6. Discussion

In this paper, we have addressed the following question: How is the average of an observable f(t,ω) affected by a weak time-dependent stimulus. We studied this question in a theoretical setting, using the linear response theory and probability distributions with unbounded memory generalizing the usual definition (3) of Gibbs distributions in statistical physics courses. Our goal was to show a general mathematical formalism allowing one to handle spike correlations as a result of neuronal network activity in response to a stimulus. The most salient result of this work is that the difference of an observable average in response to a time-dependent external stimulus of weak amplitude can be computed from the knowledge of the spontaneous correlations, i.e., from the dynamics without the stimulus. This result is not surprising from a non-equilibrium statistical physics perspective (Kubo relations, fluctuation–dissipation relation [31,33]). However, to the best of our knowledge, this is the first time it has been established for spiking neuronal networks. The novelty of our approach is that it provides a consistent treatment of the expected perturbation of higher-order correlations, going in this way, beyond the known linear perturbation of firing rates and instantaneous pairwise correlations; in particular, it extends to time-dependent correlations.

In addition, we made explicit the linear response kernel in terms of the parameters determining individual networks dynamics and neuron connectivity. We have provided an explicit example of this for a well-known class of models, the LIF model. This makes explicit the role of the neuronal network structure (especially synaptic weights) in the spiking response. As we show, the stimulus-response and dynamics are entangled in a complex manner. For example, the response of a neuron *k* to a stimulus applied on neuron *i* does not only depends on the synaptic weight $W_{ki}$ but, in general, on all synaptic weights, because the dynamics create complex causality loops which build up the response of neuron *k* [8,43,44]. Our linear response formula is written in terms of the parameters of a spiking neuronal network model and the spike history of the network. Although a linear treatment may seem a strong simplification, our results suggest that already, in this case, the connectivity architecture should not be neglected. In the presence of stimuli, the whole architecture of synaptic connectivity, history and the dynamical properties of the networks are playing a role in the correlations through the perturbed potential. This agrees well with results from a recent study exhibiting an exact analytical mapping between neuronal network models and maximum-entropy models, showing that, in order to accurately describe the statistical behavior of any observable in the Maximum Entropy model, all the synaptic weights are needed, even to predict firing rates of single neurons [8].

Higher order terms can not only play a significant role in spatial terms, as shown in [63,64], but also in temporal terms. Indeed, as argued throughout this paper, neuronal interactions involve delays that have to be integrated into a model attempting to explain spike statistics [21]. Note, however, that contrary to what is usually believed, detailed balance is absolutely unnecessary in order to properly handle time correlations [65,66,67]. Note also that binning-which can be convenient to remove short-range time-correlations from the analysis-dramatically changes the nature of the process under investigation, rendering it non-Markovian [68].

We have also introduced the monomial expansion by providing a canonical way of decomposing the potential, describing the stationary dynamics. Moreover, the Hammersley–Clifford decomposition allows us to obtain the coefficients weighting the monomials in terms of the parameters constraining dynamics. In the case of the discrete-time LIF model, this allowed us to show the explicit dependence of the coefficients in terms of synaptic weights. Although the basis of monomials is quite huge, standard results in ergodic theory and transfer matrices/operators state that we can neglect high order terms because of the exponential correlation decay.

In the example we present we study the following question: What is the role of this lateral connectivity in motion processing in sensory neurons? Clearly, one may expect it to induce spatial and temporal correlations in spiking activity, as an echo, a trace, of the object’s trajectory. These correlations cannot be read in the variations of firing rate; they also cannot be read in synchronous pairwise correlations as the propagation of information due to lateral connectivity necessarily involves delays. This example raises the question about what information can be extracted from spatio-temporal correlations in a network of connected neurons submitted to a transient stimulus.

Our results are written in terms of Kernels which can be found for any observable, generalizing the concept of receptive fields to general spatio-temporal observables beyond firing rates. This is the analogous of having “population receptive fields” which are an extension of the concept of receptive-field usually associated individual sensory neurons.

## Data Availability

The codes and instructions required to simulate spiking data and to reproduce our numerical results can be found at https://github.com/brincolab/Linear-response.

## Appendix A. Example Hammersley–Clifford Decomposition

The decomposition (6) is straightforward. However, we would like to give a simple, yet illustrative example. Let us consider 1 neuron at times 0 and 1. The spiking patterns are $\omega =(\omega_{1}\left(0\right)\;\omega_{1}\left(1\right))$=$\left(0,0\;\right)$, $\left(1,0\;\right)$, $\left(0,1\;\right)$, $\left(1,1\;\right)$, and, a function of these patterns takes four values: $f\left(0,0\;\right)=F_{0}$, $f\left(1,0\;\right)=F_{1}$, $f\left(0,1\;\right)=F_{2}$, $f\left(1,1\;\right)=F_{3}$. We have:

$$f\left(\omega \right)=F_{0}\;\left(1-\omega_{1}\left(0\right)\;\right)\left(1-\omega_{1}\left(1\right)\;\right)+F_{1}\;\omega_{1}\left(0\right)\left(1-\omega_{1}\left(1\right)\;\right)$$

$$+F_{2}\;\left(1-\omega_{1}\left(0\right)\;\right)\omega_{1}\left(1\right)+F_{3}\;\omega_{1}\left(0\right)\omega_{1}\left(1\right),$$

that we may rewrite in the form (6):

$$\begin{array}{cc} f(\omega ) & =f_{0}+f_{1}\;\omega_{1}\left(0\right)+f_{2}\;\omega_{1}\left(1\right)+\;f_{3}\;\omega_{1}\left(0\right)\omega_{1}\left(1\right);\\ f_{0} & =F_{0};\;f_{1}=F_{1}-F_{0};\;f_{2}=F_{2}-F_{0};f_{3}=F_{0}-F_{1}-F_{2}+F_{3} \end{array}$$

More generally the transformation from the “function” representation (the vector *F* of $F_{i}$s in the example) to the monomial representation (the vector *f*, of $f_{l}$s) is given by the linear transformation:

(A1) f=Q·F,

where *Q* is a triangular matrix given by [45]:

$$Q_{ll^{\prime }}=\left\{\begin{array}{c} {\left(-1\;\right)}^{d\left(l\right)-d\left(l^{\prime }\right)};\;l^{\prime }⊑l\\ 0;\;otherwise \end{array}\right.$$

Here the notation $l^{\prime }⊑l$ means the following: To each spike block $\omega_{0}^{D}$, one can associate a unique integer:

(A2) $$l=\sum_{k=1}^{N}\sum_{n=0}^{D}2^{n\;N+k-1}\;\omega_{k}\left(n\right),$$

called the index of the block. We define the block inclusion ⊑ on $\Omega_{N,R}$: $\omega_{0}^{D}⊑{\omega {\;}^{\prime }}_{-D}^{0}$ if $\omega_{k}\left(n\right)=1\Rightarrow \omega_{k}^{\prime }\left(n\right)=1$ (all bits ’1’ in $\omega_{0}^{D}$ are bits ’1’ in ${\omega {\;}^{\prime }}_{-D}^{0}$), with the convention that the block of degree 0 is included in all blocks. By extension $l^{\prime }⊑l$ means that all bits ’1’ in the block corresponding to the integer *l* are included in the block corresponding to $l^{\prime }$ [8].

The result (A1) shows that the coefficient of a monomial is the linear combination of function values where the number of terms in the combination increases with the monomial degree.

## Appendix B. Monomials Decomposition

### Appendix B.1. Linear Response in the Finite-Range Potential Approximation

Although ϕ, $\phi^{\left(sp\right)}$ have infinite range, their memory dependence can decay fast, typically exponentially. This property is independent of the application of a stimulus: it holds for ϕ and $\phi^{\left(sp\right)}$ as well, hence for δϕ. In this case, the infinite range potentials can be approximated by a finite-range one. We use here a classical result in ergodic theory: a potential ϕ with an exponentially decaying variation (11) (more generally, so-called regular potential) can also be approximated by a finite-range potential $\phi^{\left(R\right)}$ in the sup norm where $∥\phi -\phi^{\left(R\right)}∥\leq C\Theta^{R}$, for some 0<Θ<1[69]. Equivalently the chain with infinite memory can be replaced by a Markov chain of memory depth *D*, D=R+1. Therefore, δϕ can be approximated by a range-*R* potential $\delta \phi^{\left(R\right)}$. Using the monomial decomposition (6), we have:

$$\delta \phi \left(r,\omega \right)∼\sum_{l^{\prime }}\delta \phi_{l}\left(r\right)m_{l}\left(\omega_{r-D}^{r}\right).$$

In this setting, (16) becomes:

$$\delta^{\left(1\right)}\left[f(t)\;\right]=\sum_{r=n_{0}+1}^{n=\left[\;t\;\right]}\sum_{l,l^{\prime }}f_{l}\left(t\right)\;\delta \phi_{l^{\prime }}\left(r\right)\;C^{\left(sp\right)}\left[m_{l}\left(\omega_{n-D}^{n}\right),m_{l^{\prime }}\left(\omega_{r-D}^{r}\right)\;\right].$$

However, $\mu^{\left(sp\right)}$ is stationary by assumption. Hence the correlation $C^{\left(sp\right)}\left[m_{l}\left(\omega_{n-D}^{n}\right),m_{l^{\prime }}\left(\omega_{r-D}^{r}\right)\;\right]$ only depends on the two monomials $m_{l},m_{l^{\prime }}$ and on the time lag n−r, i.e., $C^{\left(sp\right)}\left[m_{l}\left(\omega_{n-D}^{n}\right),m_{l^{\prime }}\left(\omega_{r-D}^{r}\right)\;\right]=C^{\left(sp\right)}\left[m_{l}\circ \sigma^{n-r},m_{l^{\prime }}\;\right]$, that we write $C_{l,l^{\prime }}\left(n-r\right)$ to alleviate notations. Thus:

$$\delta^{\left(1\right)}\left[f(t)\;\right]\;=\;\sum_{r=-\infty }^{n=\left[\;t\;\right]}\sum_{l,l^{\prime }}f_{l}\left(t\right)\;C_{l,l^{\prime }}\left(n-r\right)\;\delta \phi_{l^{\prime }}\left(r\right),$$

so that the linear response is a linear decomposition of monomial correlations computed at equilibrium. We can write this in a more compact form introducing the *L*-dimension vectors $f\left(t\right)={\left(\begin{array}{c} f_{l}\left(t\right) \end{array}\right)}_{l=1}^{L}$, $\delta \phi \left(r\right)={\left(\begin{array}{c} \delta \phi_{l}\left(r\right) \end{array}\right)}_{l=1}^{L}$ and the matrix $C\left(n-r\right)={\left(\begin{array}{c} C_{l,l^{\prime }}\left(n-r\right) \end{array}\right)}_{l,l^{\prime }=1}^{L}$. We write $\delta^{\left(1\right)}\left[f(t)\;\right]$ in the form:

(A3) $$\delta^{\left(1\right)}\left[f(t)\;\right]\;=\;\sum_{r=-\infty }^{n=\left[\;t\;\right]}\langle f\left(t\right)|C\left(n-r\right)\cdot \delta \phi \left(r\right)\rangle ,$$

where 〈∣〉 denotes the standard scalar product.

This has three important consequences.

### Appendix B.2. The Convolution Kernel

For a time independent observable f(ω) we introduce $K_{f}\left(m\right)$ the L×L matrix with entries:

$$K_{f;l,l^{\prime }}\left(m\right)=f_{l}\;C_{l,l^{\prime }}\left(m\right),$$

so that:

$$\delta^{\left(1\right)}\left[f(t)\;\right]\;=\;\sum_{r=-\infty }^{n=\left[\;t\;\right]}\sum_{l,l^{\prime }}\;K_{f;l,l^{\prime }}\left(n-r\right)\delta \phi_{l^{\prime }}\left(r\right),$$

which is a discrete convolution. It is close to the form (1) with the difference that the time is discrete, coming from our spike trains discretization. The stimulus, explicit in (1) is here hidden in δϕ(r). We give an illustration of this in the next section. However, the fundamental result is that the convolution kernel defined this way is a linear combination of monomial correlations functions. This has, therefore, the form of a Kubo equation where the monomials play the role of the physical quantities introduced in Section 2.1. As mentioned above, the main and essential difference is that, in contrast to Physics, we have no a priori idea which monomials are the most important (except straightforward arguments on the decaying probability of high order monomials). There is no known principle to guide the choice.
